# An electroporation strategy to synthesize the membrane-coated nanoparticles for enhanced anti-inflammation therapy in bone infection

**DOI:** 10.7150/thno.48407

**Published:** 2021-01-01

**Authors:** Miusi Shi, Kailun Shen, Bin Yang, Peng Zhang, Kangle Lv, Haoning Qi, Yunxiao Wang, Mei Li, Quan Yuan, Yufeng Zhang

**Affiliations:** 1 State Key Laboratory Breeding Base of Basic Science of Stomatology (Hubei-MOST) and Key Laboratory of Oral Biomedicine, Ministry of Education, School and Hospital of Stomatology, Wuhan University, Wuhan 430079, China; 2 Key Laboratory of Catalysis and Energy Materials Chemistry of Ministry of Education & Hubei Key Laboratory of Catalysis and Materials Science, College of Resources and Environmental Science, South-Central University for Nationalities, Wuhan 430074, China; 3 Key Laboratory of Biomedical Polymers of Ministry of Education, College of Chemistry and Molecular Sciences, Wuhan University, Wuhan 430072, China; 4 Medical Research Institute, School of Medicine, Wuhan University, Wuhan, 430071, China

**Keywords:** nanoparticles, cell membrane coating, drug delivery, electroporation, nanotechnology, anti-inflammation

## Abstract

The cell membrane-coated nanoparticles (MNPs) showed great potential in treating infectious disease due to their superior biofunctions in improving biocompatibility of nanoparticles and neutralization of pathogen or toxins. However, bone infection is accompanied with severe inflammation and bone loss, which also requires anti-inflammatory and osteoconductive treatment. The conventional membrane coating method has to undergo ultrasonication and extrusion procedures, which reduces the functionality of cell membrane and limits the choice of nanoparticles. In this study, we proposed an electroporation-based membrane coating strategy to facilitate the synthesis of MNPs to tackle those problems.

**Methods:** Magnetic composite nanoparticles with osteoconductive Ca_3_(PO_4_)_2_ and bactericidal TiO_2_ were assembled into macrophages through phagocytosis and then collected to expose in electric field for obtaining macrophage membrane-coating nanoparticles. By using molecular dynamics simulation and materials characterizations, the cell membrane coating efficiency was confirmed. The *in vitro* anti-bacterial and anti-inflammatory abilities were tested by bacteria culturing and immune cells activation. Then drug-resistant bacteria induced bone infection model was established to verify its *in vivo* therapeutic effects.

**Results:** The coated membrane prepared through electroporation reserved the integrality of membrane structure and right-sidedness, with more functional proteins. Those led to the superior properties of recognition and adsorption with bacteria, toxins and inflammatory cytokines. Owing to the benefits of electroporation, the MNPs exhibited significant better antibacterial and anti-inflammatory abilities for enhancing the tissue repair process.

**Conclusion:** This study provides a novel self-assembly cell membrane coating strategy by electroporation to construct multifunctional membrane-coating nanoparticles for bone infection treatment. This strategy not only improves the functions of coated membrane, but is also proved to be universal for varies nanoparticles or cells, indicating a great potential for future applications in the bioengineering field.

## Introduction

Bone infection is one of the oldest diseases characterized by severe inflammation and progressive bone destruction, leading to fractures and sepsis if uncontrolled. The successful invasion of bacteria is highly attributed to its arsenal of virulence factors and drug resistance 1. The secreted toxins not only directly attack bone cells, but also initiate powerful immune response, producing inflammatory cytokines and enzymes to break down the infection and surrounding tissues. Followed by vascular impairment, necrotic bone (sequestrum) was formed which further made the infection area impervious to immune system and systemic antibiotics 2. The treatment of bone infection often requires anti-inflammation therapy on the basis of effective anti-bacterial process. Then, induced bone tissue regeneration with the application of degradable bone substitutes. Considering this, multifunctional nanomaterial with anti-bacterial, anti-inflammatory and osteoconductive properties shows great potential to tackle the problem.

In recent years, cell membrane-based therapies are emerging as promising platforms for promoting the biofunctions of nanomaterials to solve a great numbers of healthcare problems, including tumor diagnosis and treatment, microbial infections, and immune diseases 3-5. The extraordinary diversity of cell membrane functionalities enables the development of nanoparticle-based platforms with wide-ranging applicability 6-9. Cellular membranes from immune cells possess numerous bacteria specific cellular receptors and their sponge-like structure contributes to strong affinities with toxins and cytokines, which are beneficial for anti-bacteria and anti-inflammation therapy 10, 11. Conventional membrane coating methods are achieved through two steps: 1) preparing cell membrane vesicles; 2) extrusion of the mixture of vesicles and nanoparticles through porous membrane or sonication 3, 4. Although the conventional method can effectively coat nanoparticles with cell membrane, resultant particles from sonication can vary significantly in terms of size and lack uniformity 12, 13. Extrusion will also destroy the integrity of the membrane thus reduces the membrane's functions 14. The micromechanics of lipid membranes play a vital role on modulating the activities of the embedded functional proteins or peptides (like membrane receptors). However, the mechanical compressive force will induce structure changes in lipid mesophases, influence the kinetics of phases separation and bilayers orders, as well as the stability of lipid-protein assemblies 15-17. Moreover, application of high hydrostatic pressure leads to conformational changes of proteins ranging from molecules' compression to the loss of original structures which finally lead to protein denaturation 18, 19. These drawbacks reduce the combination with target cells and decrease the adsorption of cytokines or toxins, limiting the application for the anti-bacterial and anti-inflammatory therapy. In addition, the restriction of filter membrane pore size limits its application on larger scale bone substitutes. New technologies for better cell membrane-nanoparticles integration are required.

Among several choices in permeabilization-based disruption strategies, electroporation technique was firstly established in medical field since 1980s, and then became widely used in cell transfection 20, 21. Higher intensity of electric field directly influences the permeability of cell membrane, which allows the penetration of DNA, protein, drugs, peptides and even nanoparticles 22. Molecular dynamics simulations showed aqueous pores formed in the lipid bilayer when applying electric pulse, which is currently considered as the initial membrane perturbation 23. The leakiness of cell membrane can persist from seconds to minutes, and even hours following electric pulse application 24. After electroporation, pulsed cells still preserve certain viability under controlled parameters. Thus, such technology shows a great potential to serve for the membrane-coated strategy to leak the cytoplasm containers through the temporary pores with the maintenance of membrane functions.

**Scheme 1:**
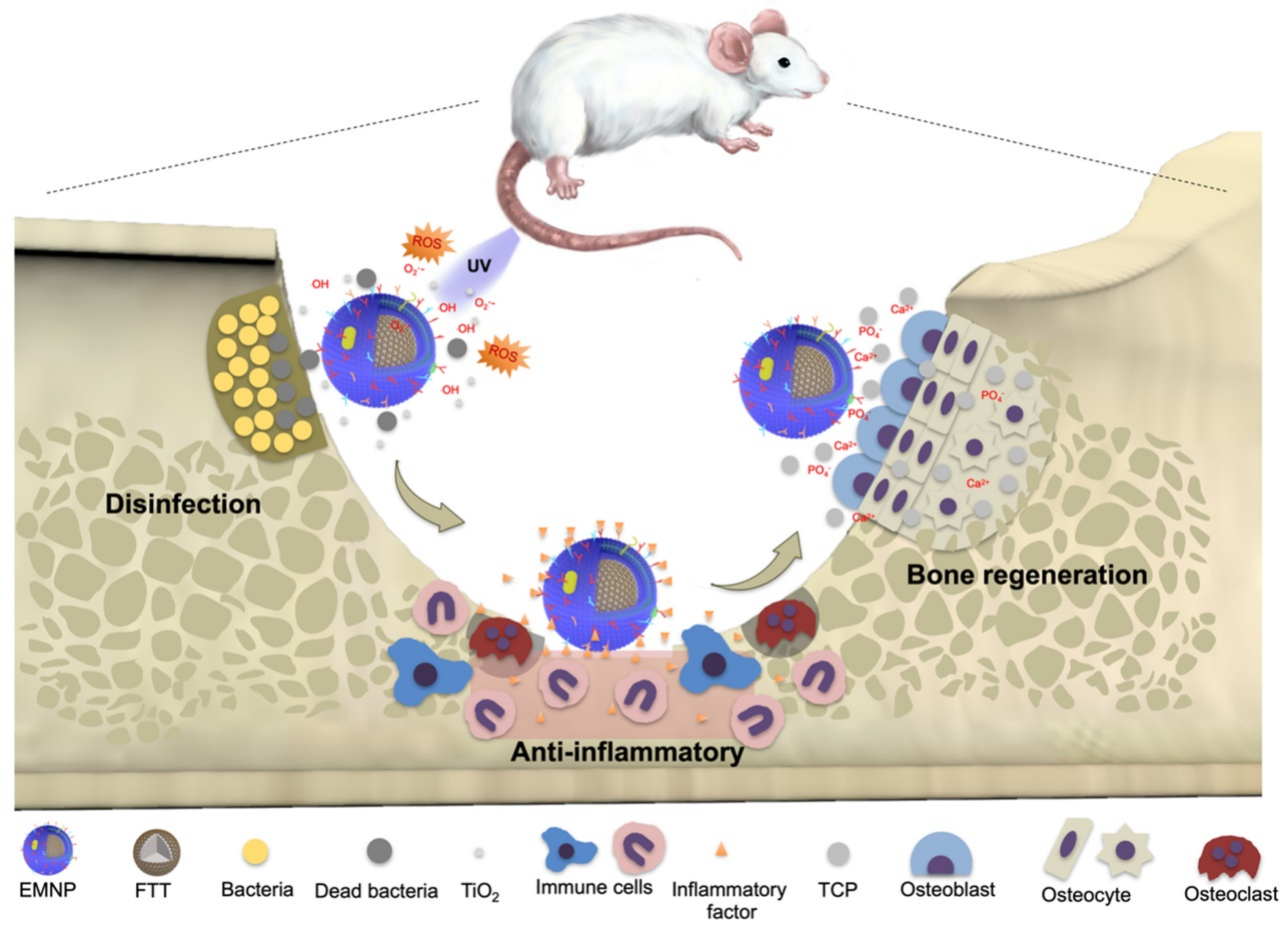
Schematic diagrams of the preparation and application of membrane-coated nanoparticles by electroporation (EMNPs). During bone infections, EMNPs could promote the bactericidal effects, inhibit the inflammation and induce bone tissue regeneration.

Herein, we introduced an electroporation based membrane-coated strategy to realize an effective and fully functional membrane coating for the treatment of osteomyelitis (Scheme 1). Ca_3_(PO_4_)_2_ was chosen to act as a bone scaffold core to conducive bone regeneration, while titanium oxide (TiO_2_) with strong photocatalytic capacity worked as the anti-bactericidal component. The magnetic composite nanoparticles with osteoconductive Ca_3_(PO_4_)_2_ and bactericidal TiO_2_ were assembled into macrophages through phagocytosis and then collected to expose in electric field. By heighten the electric pulse, the cell membrane was perforated to lead the cell substances escaping from holes, while maintaining the inner nanoparticles. With the retention of intact bacteria specific surface receptors and membrane component, the macrophage membrane-coated nanoparticles demonstrated a better combination with bacteria to exert bactericidal effect in drug resistant infections induced osteomyelitis effectively. Besides, the sponge-like membrane structure brings in a strong adsorption of inflammatory cytokines and bacteriotoxin, contributes to the anti-inflammatory effects for the following tissue regeneration process. Furthermore, the strategy presented demonstrates a broad applicability for nanoparticle functionalization, which fills up the gaps of existing membrane-coating strategies, promoting the bridges between the advantages of natural membrane components with functional nanomaterials.

## Results and Discussion

### Preparation and characterization of membrane-coated nanomaterials

**Figure 1:**
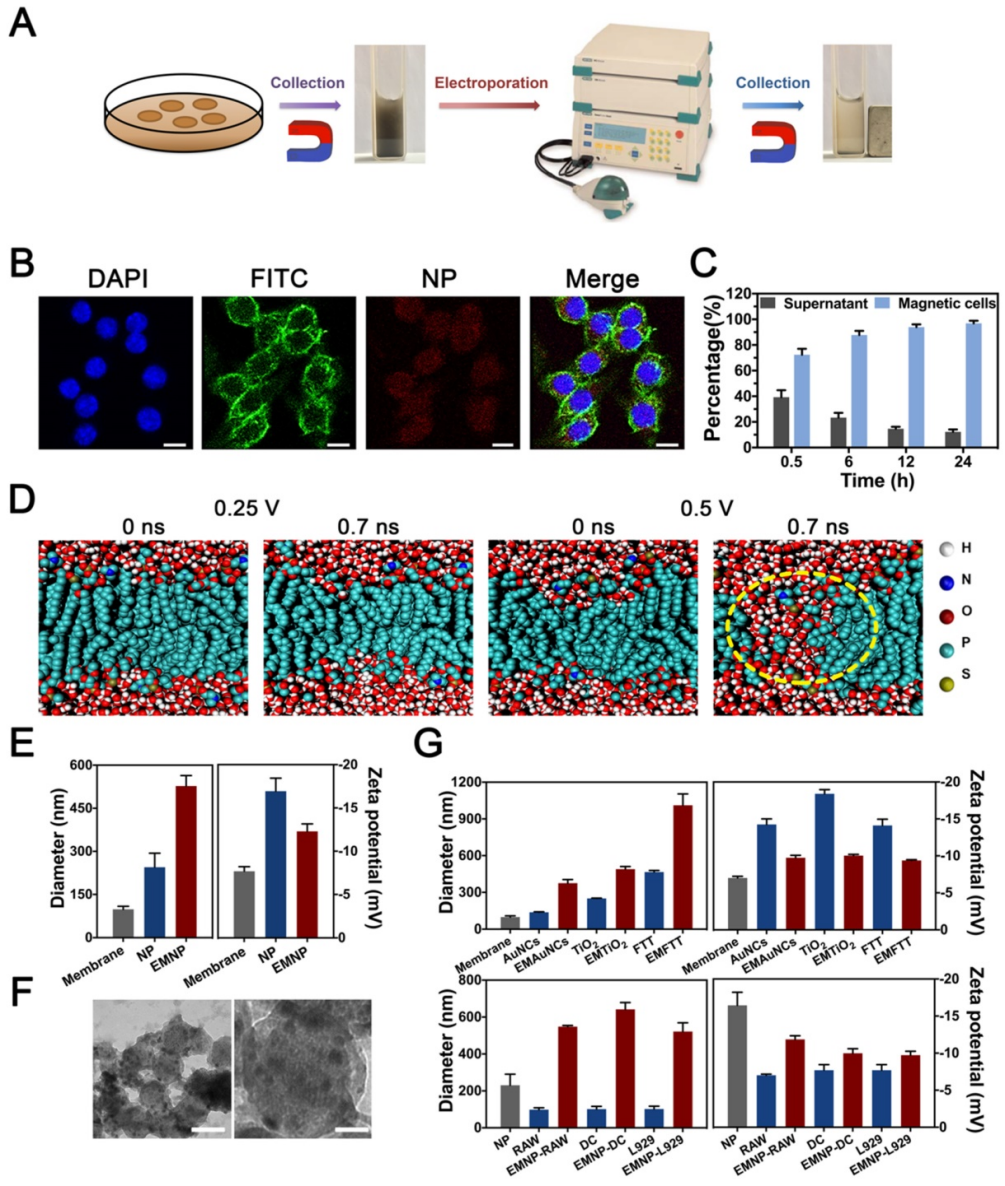
Electroporation-facilitated synthesis and characterization of membrane-coated nanoparticles. (A) Synthesis procedures diagram. (B) Fluorescence confocal microscopy image of the NPs' internalization. Blue, green and red fluorescence represented nucleus (DAPI), cytoskeleton (phalloidin) and fluorescence NPs (Rhodamine) (scale bar = 10 μm). (C) Ratio of the RAW 264.7 cells swallowed the NP during different time (0.5 h, 6 h, 12 h, 24 h). (D) Molecular dynamic simulation of DOPC biolayer under electric field of 0.25 V nm^-1^ or 0.5 V nm^-1^. Waters were shown as red and withe balls. DOPC were colored cyan balls. The yellow dotted line showed the defect of membrane structure. (E) Mean diameters and zeta potentials of NPs and EMNPs. (F) TEM images of EMNPs. (left scale bar = 100 nm, right scale bar = 20 nm). (G) Diameters and zeta potentials of obtained EMNPs from varies cells or NPs.

The preparation process of electroporation based membrane-coating nanoparticles (EMNP) consists of two major steps: the phagocytosis of nanoparticles and electroporation (Figure 1A). The composited nanoparticle was constituted from osteoconductive calcium phosphate, anti-bactericidal TiO_2_ and magnetic Fe_3_O_4_. As widely used bone substitutes, calcium phosphates could act as a natural reservoir for calcium and phosphate ions to conducive new bone regeneration 25. While TiO_2_ has great success in orthopedics and dentistry due to its benign biocompatibility, chemical stability, mechanical properties, and excellent photocatalytic capacity to release reactive oxygen species (ROS). The magnetic bactericidal nanoparticle Ca_3_(PO_4_)_2_-Fe_3_O_4_@TiO_2_ (FTT) was synthesized according to a previous report. Briefly, Ca_3_(PO_4_)_2_-Fe_3_O_4_ nanocomposites were prepared by a facile on-pot synthetic strategy using PAA-Ca template 26. Then the Ca_3_(PO_4_)_2_-Fe_3_O_4_@TiO_2_ was developed by a sol-gel method 27. FTIR spectra analysis confirmed the existence of CaP of TCP in TiO_2_@Fe_3_O_4_@TCP (Figure S1). The absorption peak at 1040 cm^-1^ indicated the P-O stretching vibration, and 570 cm^-1^, 605 cm^-1^ were attributed to the P-O bending vibration. The absorption peaks at 3424 cm^-1^ assigned to the O-H stretching vibration. X-ray diffraction (XRD) spectrum (Figure S3) showed the crystal structure of prepared TiO_2_@Fe_3_O_4_@TCP. The characteristic diffraction peaks at 25.3^o^, 37.8^o^, 48.0^o^, 53.9^o^, 62.7^o^ and 75.0^o^ are indexed to the (101), (004), (200), (105), (204) and (215) crystal planes of TiO_2_ (JCPDS No.99-0008). The characteristic diffraction peaks at 30.1^o^, 35.4^o^, 43.1^o^, 56.9^o^ and 62.5^o^ were attributed to (220), (311), (400), (511) and (440) crystal planes of Fe_3_O_4_ (JCPDS No.19-0629). It was observed that the intensity of Fe_3_O_4_ in FTT was significantly decreased that in Fe_3_O_4_@TCP with no obvious diffraction peaks of HA, represented a successful covering of TiO_2_ onto the surface of Fe_3_O_4_@TCP. TEM images further showed that the TiO_2_ layered on the surface of the Fe_3_O_4_@TCP core, constructing a nanosphere-like nanostructure (Figure S4). Such nanocomposite showed great degradability within 3 weeks (Figure S5).

After preparation of the basic nanoparticles, macrophage was chosen due to its great phagocytic capacity and abundant surface receptors 28. After co-cultured with macrophage cell line RAW 264.7 cells for 12 h, FTT nanoparticles were swallowed and internalized into RAW cells (Figure 1B). Then cells were washed with PBS to eliminate extra dissociative nanoparticles, collected and sieved by magnet to obtain nanoparticles laden cells. In order to maximize the use of cells, we cultured macrophages for different periods to find the appropriate time. After 30 minutes, 75% of RAW cells have been collected through magnet, while 90% of which was found after 6 hours. After 12 hours, there were almost 100% of cells containing NPs (Figure 1C).

Then cells were placed into an electric field to bore holes under an electroporator (Gene Pulser Xcell Electroporation System, Bio-Rad). Theoretical considerations and insights from previously reports suggested that aqueous pores were formed in the lipid bilayer during the electric pulse application 23, 29. Molecular dynamic simulation was used to test whether electric pulse could create membrane pores (Figure 1D, Figure S8). Defects appeared on the both side of DOPC bilayer at 0.7 ns after conducted with 0.5 V nm^-1^ electric field strength, while lower electric field strength failed to reach the effect.

Based on the map summarized by Weaver et al about the effects related to field strength and pulse duration 30, we chose graded conditions of voltage and verified the electroporation effects by hydrodynamic diameter and zeta potential detections. When the voltage was 300 V, the prepared membrane-coating NPs showed a smallest diameter and zeta potential (Figure S9). Since the lower or higher voltage would lead to irregular size, 300 V was chosen for the following experiments. After electrified, the size of the obtained coating FTT showed a significant increase while the zeta potential decreased slightly (Figure 1E, Figure S9 and S10). TEM visualization confirmed a relatively uniform membrane coating on the surface, demonstrating the cell membrane was successfully covered into NPs (Figure 1F, Figure S11). In addition, we repeated these procedures in varies nanoparticles and cells to confirm the universality of the electroporation method (Figure 1G). Gold nanocages (AuNCs, 100 nm), nano-TiO_2_ (250 nm) and FTT-450 (450 nm) represented nanomaterials with different diameters. Besides RAW cells, fibroblast cell line L929 cells and dendritic cell line DC 2.4 cells were also tested. Diameter and zeta potential changes of the nanoparticles proved the successfully membrane-coating effects. These results demonstrated the electroporation method could be used for varies membrane-coating demands, and the optimum conditions of electric field differed between 200~300 V according to requirements (Figure S9).

### Validation of macrophage cell membrane functionalization on EMNP

**Figure 2:**
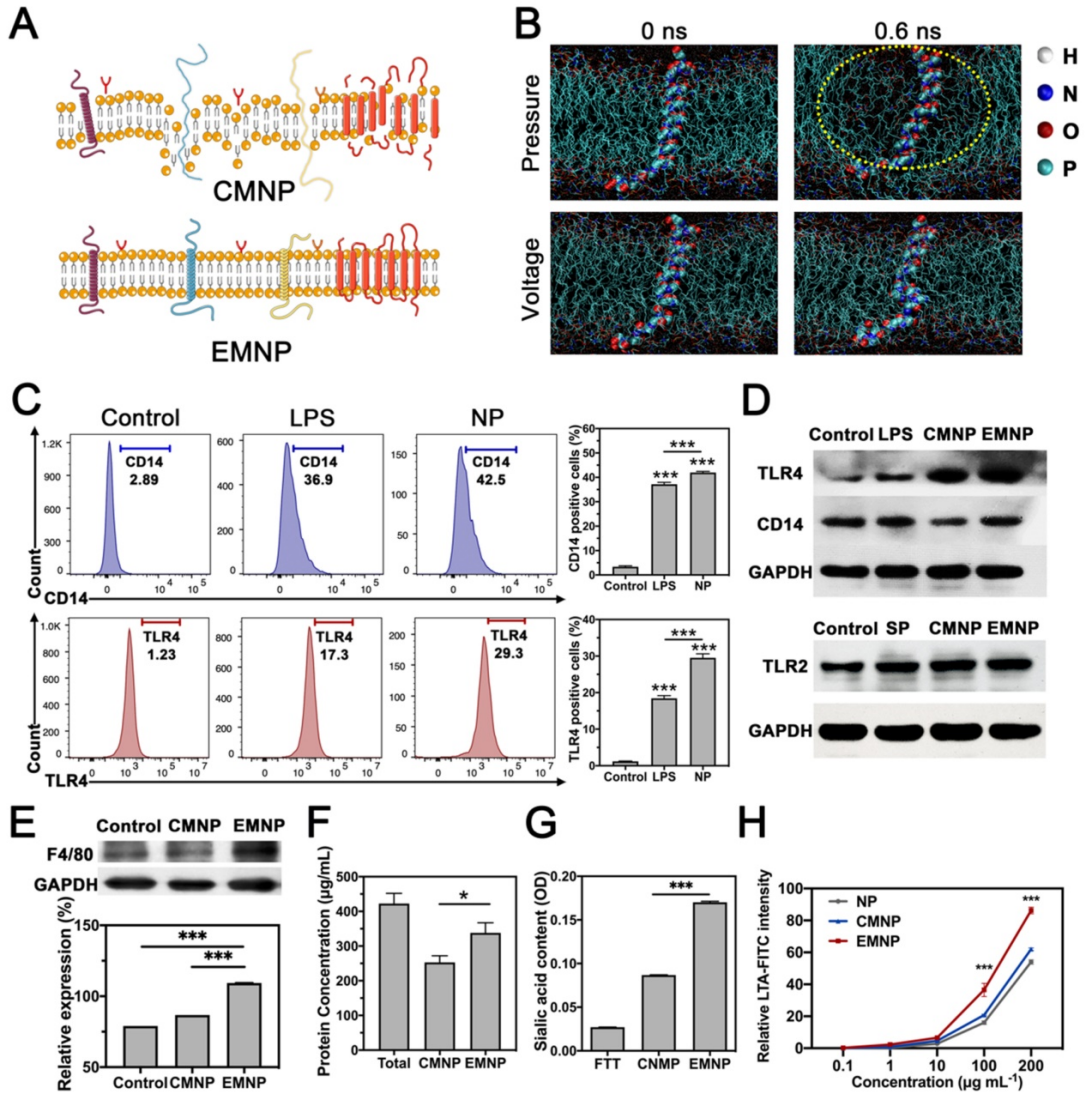
Characterization and comparison of two membrane-coated nanoparticles. (A) Illustration of the membranes changes through different coating strategies. (B) Molecular dynamic simulation of electroporation. The protein and DPPC structure changes by mechanical stress and applied electric fields. Waters were shown as red and withe balls. DOPC were colored cyan line and in the middle structure of the membrane was TLR4 protein. The yellow dotted line showed the disruption of membrane structure. (C) Flow cytometry of CD14 and TLR4 expressions in macrophages stimulated by LPS and NP. (D) Western blotting of TLR4, CD14 or TLR2 expressions in RAW 264.7 cells, cells stimulated by LPS or MRSA supernatant (SP), CMNP and EMNP after LPS or SP stimulation. (E) Western blotting and quantification of F4/80 in RAW 264.7 cells, CMNP and EMNP under same protein loading. (F) Protein concentration of RAW 264.7 cells, CMNP and EMNP. (G) Sialic acid quantification of cell membrane sidedness assay by NP, CMNP and EMNP. (H) LTA-FITC intensity detection of LTA adsorption by NP, CMNP and EMNP at different initial concentrations of LTA (*p < 0.05, **p < 0.01, ***p < 0.0001).

Then we compared the structure and functions of membrane-coated NPs fabricated by the conventional extrusion method (CMNP) and the electroporation method (EMNP). According to the molecular dynamic simulation results, the receptor proteins would be destroyed and separated from lipid bilayer after extrusion, while electroporation process kept the membrane functional structures well (Figure 2A and B).

SDS-PAGE staining of total protein showed that after membrane-coating process, the EMNP contained much more protein than CMNP under same mass loading (Figure S12). As a typical innate immune cell, macrophage employs a variety of pattern recognition receptors (PRRs) to survey danger and pathogen associated molecular patterns from environments. The detection of the bacterial endotoxin lipopolysaccharide (LPS) is highly dependent on toll-like receptor 4 (TLR4) and its co-receptor CD14 10, 31, 32. In order to test the inherit ability of the coating membrane, we used LPS to induce the pattern recognition receptors (PRRs) expression in advance. Flow cytometry experiment showed that LPS treatment effectively increase the TLR4 and CD14 expressions on macrophages. Some studies reported that calcium phosphate such as hydroxyapatite (HA) and Ca_3_(PO_4_)_2_ could induce the TNF-α production and NF-κB related inflammatory pathway in macrophage 33. Our result showed that FTT as a foreign body synergized LPS effects on TLR4 and CD14 activation, which was consistent with previous study (Figure 2C) 34. After membrane coating procedures, higher protein immunoblot intensity of TLR4 and CD14 was observed in EMNP than CMNP by Western Blot. For Gram-positive bacteria, TLR2 is highly responsible for the recognition of their microbial components. The supernatant of Methicillin-resistant *Staphylococcus aureus* (MRSA) increased the expression of TLR2 in macrophages, while after membrane-coating process, the EMNP still maintained highly expressed TLR2 (Figure 2D, Figure S13 and S14). We also detected the content of macrophage specific membrane marker EGF-like module-containing mucin-like hormone receptor-like 1 (EMR1, also known as F4/80) on MNPs. When using equivalent protein loading, the amount of F4/80 was expressively higher than CMNP and cell lysate, indicating the enhancement of membrane specific protein in EMNP (Figure 2E). The protein concentration measurement also showed that the membrane protein utilization rate of EMNP was higher than CMNP (Figure 2E and F).

Besides, lipid compositions of cell membranes are prominently different. In order to test the sidedness of the prepared MNPs, we quantify the sialic acid, a widely expressed binding ligand on immune cells. It was found that sialic acid content on EMNPs was 60% more than CMNPs (Figure 2G). This result suggested a higher ratio of right side-out membrane orientation on MNPs. However, CMNPs with inside-out membranes will be quickly cleared in the circulation, induce off-target biotoxicity and even stimulate unexpected immune responses 35.

Primary attachment is the first step of colonization when bacteria accessed the bone surface. Colonization can occur through direct interaction with bone cells, plasma proteins or ECM. Competitive binding capacity of bacteria through cell membrane on MNPs contributes highly to effectively prevent the invasion of bacteria. To confirm the recognition and bonding capabilities of EMNP on microbial membrane components, we used FITC conjugated lipoteichoic acid (LTA, a Gram-positive bacteria cell wall polymer) or LPS (an endotoxin from Gram-negative bacteria outer membrane) according to previous reports and detected the fluorescence intensity on nanoparticles 36.

**Figure 3:**
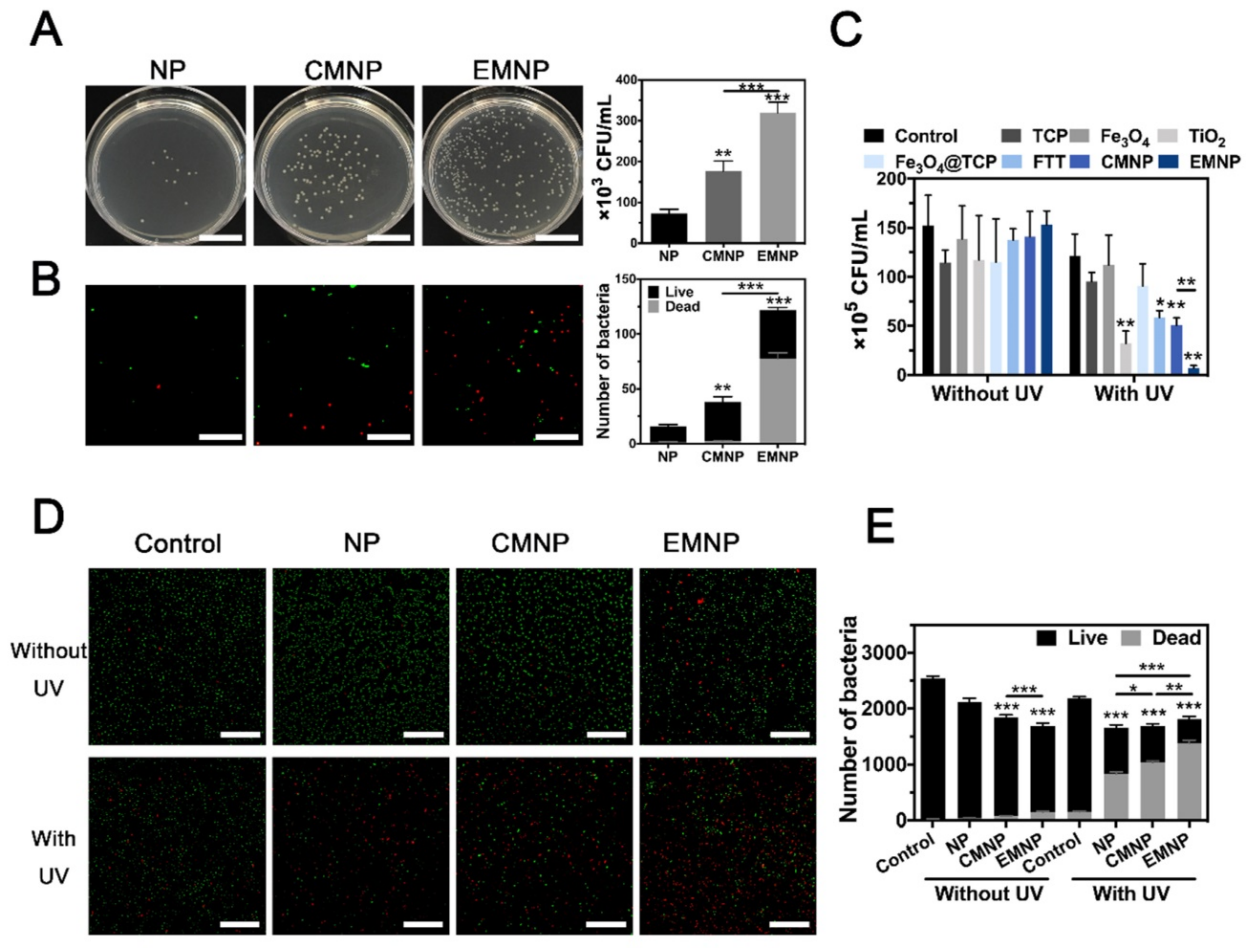
Antibacterial activity of EMNP *in vitro*. (A) Bacteria adsorption ability in plate and fluorescence microscope (B) by NP, CMNP and EMNP, and their quantification (scale bar in A = 2 cm, scale bar in B = 2 μm). (C) Quantitative analysis of bacterial colony of MRSA incubated with TiO_2_, Fe_3_O_4_, TCP, Fe_3_O_4_@TCP, TiO_2_@Fe_3_O_4_@TCP, CMNP and EMNP with or without ultraviolet irradiation (1 W cm^-2^, 5 min). (D) Live/dead staining images of MRSA incubated with NP, CMNP and EMNP with or without ultraviolet irradiation (1 W cm^-2^, 5 min, scale bar = 5 μm). (E) Quantitative analysis of Live/Dead staining (*p < 0.05, **p < 0.01, ***p < 0.0001).

As shown in Figure 2H and Figure S15, LPS-FITC adsorbed on NPs enriched with the increasing of LTA/LPS. However, the EMNP showed a robust increasing in FITC intensity than CMNP, which had significantly stronger adsorption ability at high LTA/LPS concentrations. LTA is the primary TLR2 ligand in the early phase of Gram-positive bacterial infection, the high affinity of EMNP with LTA helped neutralizing LTA activated TLR2-related inflammatory responses. While the LPS also accounts for much of the strong inflammatory responses caused by mixed bacterial infections 37, 38. The enrichment of PRRs on EMNP could bring on a superior combination of bacteria and clearance of the deleterious endotoxin. Colony-forming unit counting and cell staining further confirmed the preferential targeting ability of EMNP on bacteria (Figure 3A). After incubated with *Staphylococcus aureus (S. aureus)*, nanoparticles were collected by magnet and quantified for the adsorbed bacteria. As shown in fluorescent images (Figure 3B), *S. aureus* incubated with EMNP showed nearly 4 times increase in bacteria number than CMNP group, and even tenfold increase compared to uncoated NPs. Notably, the dead bacteria accounted for more than 60% of adsorbed bacteria in EMNP group, which demonstrated a certain bactericidal effect of EMNP along after cultured overnight.

### Antibacterial ability in vitro

After confirming the occurrence of a prominent binding between bacteria and EMNP, we proceeded to examine whether the prepared membrane-coating nanoparticles could exert superior performance on bactericidal activity. Three different types of bacteria were selected: *S. aureus* as the representative of Gram-positive bacteria, *Escherichia coli* (*E. coli*) of Gram-negative bacteria, and MRSA represent the drug-resistance bacteria. Ampicillin and vancomycin were served as negative and positive controls to combat MRSA infection. Results showed that only high dose of ampicillin (100 μg mL^-1^) could effectively reach >90% killing efficiency, while vancomycin showed excellent antibacterial effect under lower concentration (Figure S16 and S17). The standard plate counting assay showed that after treated with TiO_2_, FTT, CMNP and EMNP under 1.0 W cm^-2^ ultraviolet (UV) irradiation for 5 min, the survival of live MRSA decreased significantly (Figure 3C and Figure S18). As a nuclear and chromosome counterstain, propidium iodide (PI) can't permeant into viable cell membrane and is widely used as a dead cell indicator. After exposed to UV irradiation, 75% of EMNP treated bacteria were stained by red-fluorescent PI, suggesting the destroy of bacteria membrane by EMNP, represented the best antibacterial rate which was consonant with the results from standard plate counting assay (Figure 3D, E). These results confirmed the excellent bactericidal ability of EMNP, attributing to the macrophage membrane and ROS-produced TiO_2_ component. Likewise, results of *S. aureus* and* E. coli* were consistent with the MRSA results, in which the killing efficacy were up to 90% (Figure S19 and S20).

### Anti-inflammation ability in vitro

**Figure 4:**
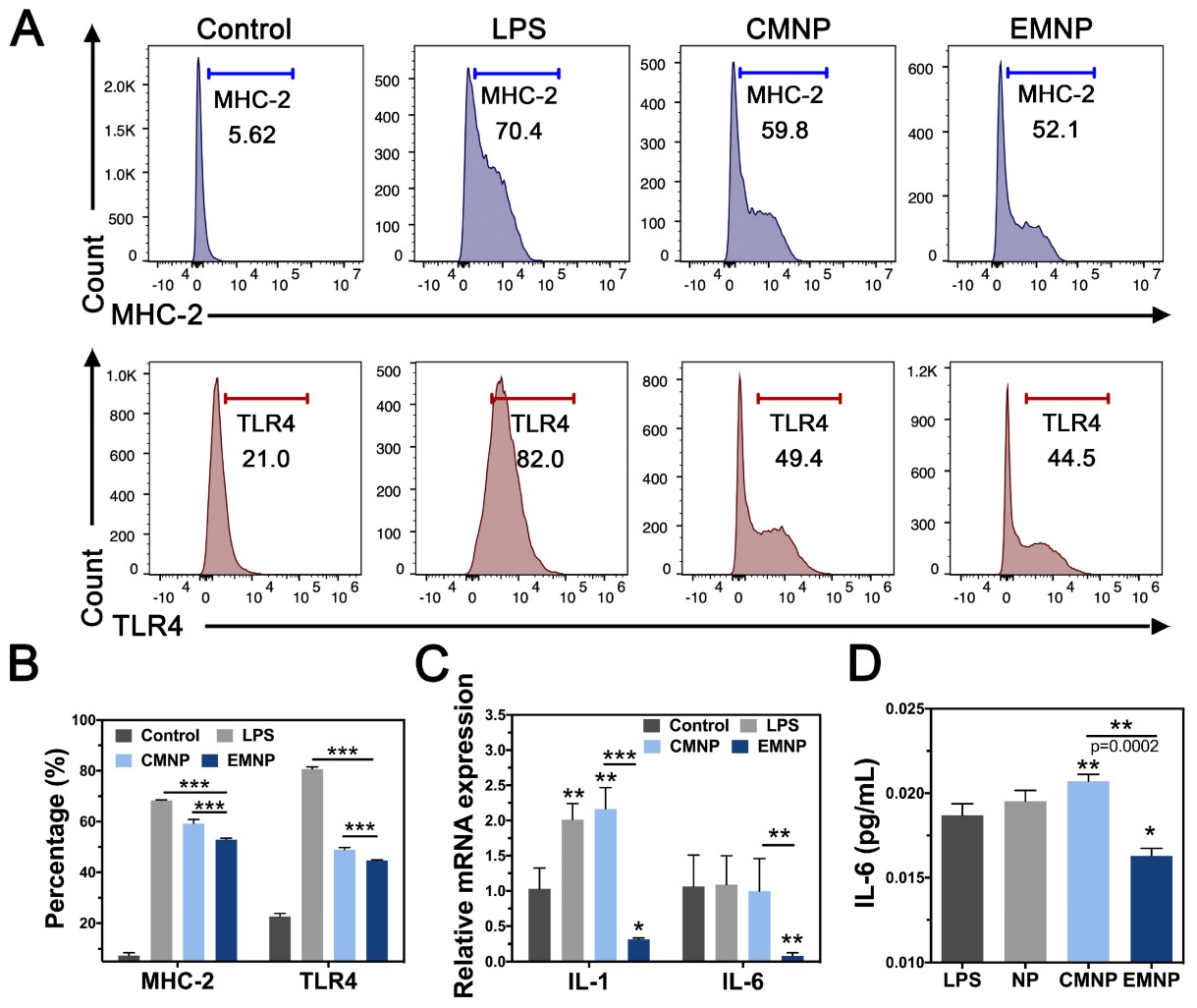
Anti-inflammatory activity of EMNP *in vitro*. (A) Flow cytometry of CD14 and TLR4 expressions in RAW 264.7 cells stimulated by LPS, CMNP and EMNP. (B) Quantitative analysis of Flow Cytometry. (C) Quantitative real-time PCR of mRNA expression of IL-1 and IL-6. (D) Quantitative analysis of IL-6 by ELISA (*p < 0.05, **p < 0.01, ***p < 0.0001).

During bone infections, besides the direct invasion of bacteria and their toxins, bacterial infection can also elicit bone resorption through host immunity. Pathogen-associated molecular patterns (PAMPs) interact with TLRs on several cell types, stimulating the release of chemokines and inflammatory cytokines such as TNF, IL-1 and IL-6. On the one hand, these cytokines lead to the production of RANKL by osteoblast, which activates osteoclast differentiation through shifting RANKL/OPG ratio 39. On the other hand, released cytokines recruit and activate more innate and adaptive immune cells, magnifying the immune responses and producing more inflammatory cytokines, which in turn facilitate bone resorption process 40, 41. As a typical effector cells, macrophage cells membrane can not only adsorb endotoxin, but also receive inflammation cytokines 42. Neutralization of inflammatory cytokines could effectively prevent neutrophil recruitment and inhibit inflammation induced tissue destruction 43, 44. In order to measure the anti-inflammatory effect of EMNP, we stimulated RAW 264.7 cells with LPS and detected the inflammatory receptors activation by flow cytometry after cocultured with MNPs. LPS strongly activated the major histocompatibility complex-2 (MHC-2) expression, a class of canonical inflammatory molecules normally expressed to initiate immune responses, while the MNPs decreased the expression significantly (Figure 4A and B). Compared with 59.8% in CMNP group, the EMNP treatment suppressed the MHC-2 expression (52.1%). Real-time qPCR detection on inflammation related genes interleukin-1 (IL-1) and interleukin-6 (IL-6) further confirmed the anti-inflammatory ability of EMNP (Figure 4C). We attributed this to the adsorption capability of macrophage membrane structure, and was supported by the reduction of the IL-6 in the EMNP treated culture medium using ELISA assay (Figure 4D).

### In vivo therapeutic potential on bone infection

**Figure 5:**
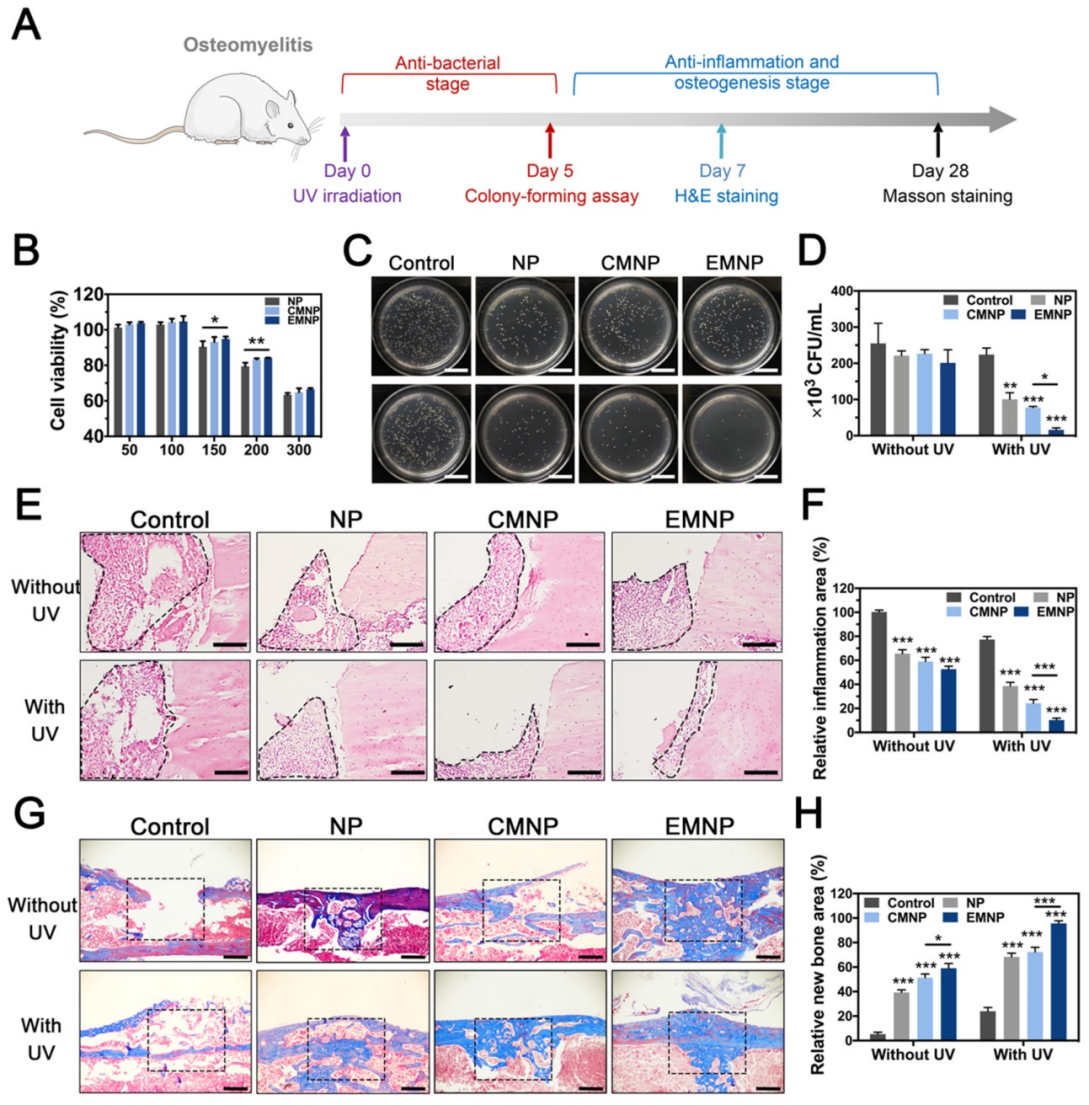
The *in vivo* therapeutic abilities of EMNP in osteomyelitis model. (A) Illustration of osteomyelitis treatment process. (B) Biocompatibility of EMNP detected by CCK-8 assay. (C) Photos and (D) quantification of the bacterial colony of the femur bone tissues treated with NP, CMNP and EMNP with ultraviolet irradiation after 5 days (scale bar = 2 cm). (E) H&E staining of inflammatory area from femur after treatment in 5 days (black dotted line showed the inflammatory infiltration area). (scale bar = 50 μm). (F) Quantitative analysis of the immune cells numbers in inflammatory sites. (G) Masson staining of bone repairing area after 4 weeks (black dotted box showed the origin bone defect area, scale bar = 100 μm). (H) Quantitative analysis of new bone area of black dotted line in (F) (*p < 0.05, **p < 0.01, ***p < 0.0001).

In order to further confirm the therapeutic potential of electroporation membrane-coating NPs *in vivo*, we selected drug-resistant bacteria (MRSA) induced osteomyelitis as the model (Figure 5A) 45-47. Toxicity was firstly evaluated by CCK-8 assay and EMNP exhibited a well biocompatibility during working concentration (below 150 μg mL^-1^) (Figure 5B). Histological evaluation of *in vivo* application also showed no significant differences in organs of the mice treated with different nanomaterials (Figure S22). Owing to the ability to adhere bacteria* in vitro*, EMNP was considered to be more specific binding to bacteria at the infection site *in vivo* to exhibit a local treatment effect under UV light. As expected, bacteria counting of infected bone tissues from EMNP treated mice demonstrated a much better antimicrobial activity compared to other groups 5 days after infection, equivalent to vancomycin treatment (Figure 5C and D, Figure S23). Combining the bactericidal effect of photocatalytic antibacterial TiO_2_ with high absorbability membranes, EMNP could relieve the tissue damage caused by bacteria infection and toxins attack. H&E staining showed the degree of inflammatory infiltration (Figure 5E and F). The invasion of MRSA induced a significant inflammatory cell infiltration with monocytes and foreign body giant cells gathering. By contrast, the MNPs showed a decreased accumulation of immune cells, especially in EMNP group, further proved that the induction of macrophage membrane could suppress the inflammation caused by bacteria.

Besides the microbial invasion, the excessive immune response initiated by bacteria could also boost the cell and tissue necrosis which prevents the tissue repairing process. Histological staining was conducted to analyze the long-term efficacy of tissue repair after 4 weeks. With the outstanding osteoconductive ability of TCP, new bone formation will occur simultaneously with the TCP degradation 48. Owing to the superior anti-inflammatory ability of EMNP, the bone regeneration degree was far ahead than other groups (Figure 5G and H). Thus, this membrane-coated strategy can effectively enhance the antibacterial and anti-inflammation properties, promoting the bone tissue regeneration and further healing drug-resistant bacteria induced bone infections.

## Conclusion

In summary, we presented a self-assembly cell membrane coating strategy by electroporation. After nanoparticles were internalized by phagocytosis, cell membrane will wrap onto the nanoparticles automatically under appropriate electric stimulation. By molecular dynamics simulation, the preparation method was shown to bore holes on the cell membrane without damaging the structure of biological membrane and proteins. The detection of receptors expression on the prepared EMNPs demonstrated the improved reservation of receptors numbers and activities compared with traditional method which could lead to membrane structure disruption and protein denaturation. On account of these superiorities, the EMNPs exhibited strong capabilities to recognize bacteria, adsorb bacteriotoxin and inflammatory cytokines, leading to an enhanced antibacterial and anti-inflammatory abilities both *in vitro* and *in vivo*. Drug-resistant bacteria infection model further confirmed the efficiency of EMNPs for anti-infection therapy and tissue regeneration. In addition, we investigated the effects of the electroporation method with different types of cells or nanoparticles, and provided recommended conditions respectively.

The novel strategy presented here shook off the constraints of nanoparticles size due to the filters limitation in extrusion method, without the additional equipment except the commonly used experimental instrument electroporator in cell biology, definitely reduced the difficulty and complexity for membrane coating process. The method provided here fills the gaps of the existing membrane-coating strategies, offering an inspiration for the application of biogenic nanoparticles in biomedical field.

## Materials & Methods

### Materials synthesis

To synthesize TiO_2_@Fe_3_O_4_@TCP nanoparticles, Ca_3_(PO_4_)_2_-Fe_3_O_4_ was firstly prepared by a facile on-pot synthetic strategy using PAA-Ca template 26. Briefly, 60 mg calcium hydroxide was dispersed in DI water (100 mL) under ultrasound. Then, polyacrylic acid (PAA, 1 mL 0.2 g mL^-1^) was added with magnetic stirring to obtain the complex of polyacrylic acid and Ca^2+^ ion complex (PAA-Ca). After 30 min stirring, isopropyl alcohol (IPA, 200 mL) was added to obtain PAA-Ca nanocapsules. After stirred for another 2 h, iron (II) chloride tetrahydrate (98%) (100 mg FeCl_2_ in 0.2 mL DI water) was added to obtain PAA-Ca/Fe(OH)_3_ nanospheres. Diammonium hydrogen phosphate (71 mg of (NH_4_)_2_ HPO_4_ in 0.2 mL DI water) was added four times with 30 min intervals within 2 h. The solution was continued stirring for 12 h at room temperature to obtain PAA/CaP/Fe(OH)_3_ nanoparticles and then collected by centrifugation at a speed of 8000 rpm for 5 min. After washed with DI water and anhydrous ethanol until neutral, the PAA/CaP /Fe(OH)_3_ NP was dried at 60 °C in the oven. Then put the powder in a muffle furnace and calcined at 600 °C (heating rate at 5 °C min^-1^) for 6 h under nitrogen atmosphere. The collected black magnetic powder was Ca_3_(PO_4_)_2_-Fe_3_O_4_ magnetic composite microspheres, namely Fe_3_O_4_@TCP.

The TiO_2_ collosol was prepared by a sol-gel method. 1.2 mL fuming nitric acid was diluted by DI water to 200 mL, and transferred into a 500 mL round-bottomed flask. After adding tetrabutyltitanate solution (29.0 g tetrabutyltitanate dissolve in 10 mL anhydrous ethanol) under stirring, the reaction system was heated to 180 °C and refluxed under oil bath for 8 h to get TiO_2_ sol. Then 0.2 g Fe_3_O_4_@TCP was ultrasonically dispersed in 50 mL anhydrous ethanol and added with TiO_2_ sol and stirred for 3 h. Samples was collected by a magnet then was washed three times with anhydrous ethanol. After drying at 180 °C, magnetic Ca_3_ (PO4)_2_-Fe_3_O_4_@TiO_2_ microsphere was collected, namely TiO_2_@Fe_3_O_4_@TCP (FTT).

### Materials characterization

The magnetic testing was obtained by using the magnet. FTIR spectroscopy spectra were performed by a Nicolet Co NEXUIS-470 FTIR spectrometer. XRD patterns were measured by XRD, X' Pert Pro, Philips, the Netherlands. UV-vis absorption spectroscopy was carried out by UV-Vis spectrophotometer (UV-2550, Shimadzu, Japan).

The ROS release of the TiO_2_@Fe_3_O_4_@TCP was examined by the degradation of Rhodamine B. Mixed the TiO_2_@Fe_3_O_4_@TCP (0.5 mL, 100 μg mL^-1^) with Rhodamine B (0.5 mL, 0.08mg mL^-1^) in tubes and treated with or without ultraviolet irradiation at 1 W cm^-2^ for different time (0 min, 10 min, 20 min and 30 min). The UV spectra of supernatants were examined by Power Wave XS2 (Bio-Tek, USA).

### Cell membrane coating

Mouse macrophage cell line RAW 264.**7**, dendritic cell (DC 2.4 cells), fibroblast cell line L929 (China Center for Type Culture Collection, CCTCC) were used for membrane-coated cells in the experiment. RAW 264.7 cells and L929 cells were cultured in DMEM with 10% fetal bovine serum (FBS, #10099141, Gibico, Australia) and 1% penicillin/streptomycin (P/S, #SV30010, HyClone, Thermo Fisher). DC 2.4 cells were cultured in RPMI-1640 medium (with 10% FBS and 1% P/S) at 37 °C in a humidified 5% CO_2_ atmosphere.

Nanoparticles were incubated with 7 × 10^6^ RAW 264.7 cells for different time points. After washed with PBS to eliminate extra nanoparticles, NP-containing cells were collected by cell scraper and further filtrated by a magnet. The cells were put into cuvettes and gave electric pulse under an electroporation system (Gene Pulser Xcell Electroporation System, Bio-Rad). Afterwards, nanoparticles were coated and collected and washed by a magnet to wipe off cell fragments. By inductively coupled plasma-atomic emission spectrometry (ICP-AES) measurement, the concentration of Fe in 100 μg mL^-1^ FTT and EMNP was 32 and 21 μg mL^-1^, respectively. Thus the final concentration of FTT in EMNP was around 65.6 μg mL^-1^. In order to test the feasibility on other cells and nanoparticles, gold nanocages (AuNC), TiO_2_ nanoparticles and larger FTT particles were used, and L929 cells, DC cells were chosen to represent different types of cells.

As comparison, the conventional membrane coating procedure was conducted by extrusion method. Briefly, RAW 264.7 cells were collected and disrupted using ultrasound. After centrifugation at 4000 rpm, the supernatants were stored, while the sediment was re-suspended in PBS and subjected to ultrasound and centrifuged again. After centrifuged the pellets at 20,000 g for 30 min, collected the supernatant and used an ultra-speed centrifuge (LE- 80K, Beckman Coulter, USA) at 25,000 rpm for 2 h. Then the pellets containing membranes were collected as purified macrophage cells membranes. For membrane coating, 150 μg NPs (in PBS) was mixed with the prepared macrophage cells membrane and repeatedly extruded through a 400 nm polycarbonate membrane (Millipore) using a mini extruder (Avanti Polar Lipids, USA). Collected the sediment by the magnet to remove excess membranes.

### Molecular Dynamics Simulation

Molecular dynamics simulations were performed to analyze pore formation by using GROMACS 5.1.25. The phospholipid bilayer was composed of phospholipid molecules and interspersed membrane proteins. Here, the combination of GROMOS and Berger force field were used to simulate dipalmitoyl-phosphatidylcholine (DPPC) and TLR4 transmembrane domain 49. The formation of water-filled pores was observed at atomic resolution 50. Building a bilayer which is composed of two leaflets of DPPC. The system was composed of 128 lipids and 3655 water molecules. To minimize the energy of the system by chose the steepest descent method. After minimization, the system was equilibrated by 100 ps NVT and 1 ns NPT. Then, the system was simulated with 0.25 and 0.5 V nm^-1^ electric fields at 310 K and zero surface tension. And a 1.5 nm Coulomb cutoff plus Particle-Mesh-Ewald (PME) method was used for long-range interactions 51. Molecular dynamics simulations were performed for 2.0 ns. In the second part of the simulation, the structured of a bilayer consist of 128 lipids and a TLR4 transmembrane domain 52. 3331 water molecules were added to solvate the bilayer while 2 Cl ions were used to neutralize the system. After equilibrations, a 0.1 nm ps^-2^ acceleration was applied to the system as anisotropic pressure, at 310 K with a 1.5 nm Coulomb cutoff. Molecular dynamics simulations were performed for 1.4 ns with all else conditions same as previous simulations.

### Membrane coating assessment

*Fluorescence Confocal Microscopy.* For NP's internalization observation, RAW 264.7 cells were cultured and added with the Rhodamine B-loaded NPs for 6 h. After fixing with 4% paraformaldehyde (PFA), cells were stained with DAPI and phalloidin. For EMNP observation, DAPI stain and DIO (3,3′-dioctadecyloxacarbocyanine perchlorate) stain were used to stain the nuclei and cell membranes. Images were captured by a confocal laser scanning microscope (CLSM 510 Meta, Zeiss). Excitation and optical emission filters: (DAPI, 405 nm); (FITC, 488 nm); (Rhb-NP, 552 nm).

*Size and Zeta potential.* Samples were diluted in ultrapure water to 0.01 mg mL^-1^ and then measured by (DLS, Zetasizer Nano ZS90, Malvern Instruments Ltd, UK) equipped with a 4 mW He-Ne laser (λ = 633 nm) and a scattering angle of 90° at room temperature.

*Transmission Electron Microscope (TEM) scanning.* NP or EMNP dispersions were dropped onto carbon film-coated 300-mesh TEM copper grids. After dried at room temperature, samples were characterized under a transmission electron microscope (TEM, 200 kV, Tecnai G2 20, FEI Corp, Netherlands).

*Flow Cytometry.* RAW cells were cultured in 6-well plate at a density of 1 × 10^6^ pretreating with LPS (100 ng mL^-1^) for 12 h and then cultured with NP for 6 h. Cells were collected by the magnet and stained with PerCP-Cy5.5 CD14 (#560638, BD Bioscience, USA) and PE/Cy7-CD284 (TLR4) (#B234490, Biolegend, USA) at 4 °C for 30 min. After wash centrifugation, the expressions of CD14, TLR4 receptor were calculated by a flow cytometer (Beckman Coulter, USA). Data were analyzed via FlowJo software.

*Protein utilization.* Membrane-coated materials were collected by the magnet while RAW cells membrane was also collected as the total protein content. Added 100 μL RIPA buffer to split the protein and followed by the BCA assay to quantify the protein amount. The ratio of utilization and loss was calculated by the OD values of the treated groups (T) and the OD values of the control (C) (T/C × 100%). Then 10 μg proteins were loaded for SDS-PAGE and the gel was stained for 2 h in Commassie Blue followed with washing for observation.

*Western blot.* The identification of membrane receptors expressions was performed using primary antibodies including rabbit anti-mouse TLR4 (ABclonal, A0007) and rabbit anti-mouse CD14 (Boster, PB0893), rabbit anti-mouse TLR2 (ABclonal, A11225) and rabbit anti-mouse EMR1 (F4/80, ABclonal, A1256). Proteins from CMNP, EMNP were prepared followed the previous process. Following SDS-PAGE, proteins were transferred onto blotting membrane and blotted in milk. Then rinsed the blot and incubated with primary antibodies overnight at 4 °C. Then incubated HRP Goat anti-mouse IgG (ABclonal, AS003) for 1 h at room temperature. After washing by 1 × TBST, protein bands were visualized by Luminous fluid (Thermo Fisher, USA).

*Sialic acid assay.* The sialic acid assay was conducted according to a previous report 53. NP, CMNP or EMNP were mixed with 0.2 mL 0.05 N sulfuric acid respectively for 15 min in boiling-water bath and cooled down. Then incubated in 0.2 mL 10 mmol L^-1^ periodate reagent at 37 °C for 30 min. Eliminated the excess periodate by adding 0-1 mL 35 g L^-1^ sodium thiosulfate until the yellow color of the periodate has disappeared. Immediately added 2 mL of the thiobarbituric acid reagent and heated in water bath for 10 min. After cooling down, the solutions were shaken with 2 mL of the acid butanol. The separation of the two phases was rapidly centrifuged, and the intensities of the colors in the butanol layer were compared at 549 nm.

*LTA/LPS adsorption ability.* To determine the adsorption capability of EMNP, the fluorescein isothiocyanate (FITC) conjugated LTA or LPS was chosen 36. In brief, 4 mg LTA or LPS was made monomeric by treatment with 2 mL of 0.5% trimethylamine (Sigma, USA). After sonication, EDTA (200 μL, 100 mM) was added. Then added 20 mg FITC and sonicated for 1 min. After adding 1 mL 1.6% sodium deoxycholate (Sigma, USA) was incubated for 18 h at 37 °C while rotating. After that, the LTA/LPS-FITC was diluted to 0.1 μg mL^-1^, 1 μg mL^-1^, 10 μg mL^-1^, 100 μg mL^-1^ and 200 μg mL^-1^ and combined with NP, CMNP or EMNP for 30 min. Then the mixture was collected by the magnet. After discarding the supernatant, the sediment was added into 96-well plates to test the fluorescence intensity by Spectramax i3x.

*Bacteria adsorption ability.* Gram-positive bacteria *Methicillin Resistant Staphylococcus Aureus* (MASA)(ATCC 43300),* Staphylococcus aureus* (ATCC BAA-1721) and Gram-negative bacteria *Escherichia coli* (ATCC 43888) were grown in LB broth at 37 °C. After measured the optical density at 600 nm (OD 600 nm), bacterial suspensions were diluted to 5 × 10^6^ colonies forming units (CFU per mL) for following experiments. To investigate the ability of NP, CMNP and EMNP on bacteria adsorption, 500 μL MRSA were combined with 500 μL 100 μg NP, CMNP or EMNP. After 30 min, the mixture was collected by the magnet. After discarding the supernatant, 25 μL sediment was spread onto LB agar plates respectively. For live/dead staining, the sediment was washed with PBS three times and stained with PBS containing 2 μM calcein-AM and 1 μM propidium iodide (PI) for 30 min. Images were taken using a Zeiss Axio Imager A2 fluorescence microscope and quantitative analysis of live and dead bacterial cells was performed using Image J software.

### Antibacterial properties

To investigate the antibacterial ability of different materials, 20 mL MRSA were prepared in liquid LB medium at 5 × 10^6^ CFU mL^-1^. Then 500 μL bacterial liquid were supplemented with 500 μL NP, CMNP, EMNP (150 μg), and PBS, ampicillin or vancomycin as a control group. After that, the liquids were treated with or without ultraviolet irradiation (1 W cm^-2^) for 5 min. The standard plates counting was performed 1 h after irradiation treatment while rotating, 25 μL bacterial suspension was spread onto LB agar plates respectively. The plates incubated in the incubator at 37 °C with 95% humidity and 5% CO_2_ for 24 h.

For live/dead staining, 200 μL 1 × 10^7^ CFU mL^-1^ MRSA cells were added onto a glass coverslip and cultured for 2 h. After wash with PBS, new LB broth were added for another 24 h to obtain bacterial biofilm. Then the biofilms were immersed in 0.5 mL PBS, NP, CMNP and EMNP at 150 μg mL^-1^ for 2 h, then washed three times and treated with or without ultraviolet irradiation (1 W cm^-2^) for 5 min. The biofilms were stained with calcein-AM and PI as described before.

To investigate the effect of three different materials on the bacterial growth rate, 500 μL MRSA (5 × 10^6^ CFU mL^-1^) were added with NP, CMNP and EMNP at a final concentration of 150 μg mL^-1^. After that, the liquids were treated with or without ultraviolet irradiation (1 W cm^-2^) for 5 min. Bacterial concentrations were measured every hour at 600 nm until 24 h.

### In vitro anti-inflammatory abilities

The *in vitro* biocompatibility was first investigated by culturing RAW 264.7 cells with different concentrations of NPs, CMNP, and EMNP (50, 100, 150, 200, and 300 μg mL^-1^). After grew for 24 h and washed, CCK-8 assay was measured at OD 450 by a microplate reader (Emax Precision, USA). Then RAW 264.7 cells were seeded at 1 × 10^4^ cells per well in 24-well plates and pretreated with LPS (100 ng mL^-1^) for 12 h then incubated with MNPs for 6 h. Then total RNA was isolated using TRIzol reagent (Invitrogen). After quantifying the RNA concentration by Nanodrop2000 (Thermo Fisher Scientific), 1 μg RNA was used for reverse transcription (TaKaRa, Japan). CFX Connect™ Real-Time PCR Detection System (Biorad, USA) was used to quantify the real-time PCR cycles. The sequences of primers for IL-1, IL-6, were listed in Table S1. Gene expression levels were calculated by ΔΔCt method normalized to GAPDH and calibrated to control samples which were RAW without treatment.

For flow cytometric analysis, cells were collected and stained with PE IA-IE (MHC II, #107629, Biolegend, USA) and PE/Cy7-CD284 (TLR4) (#B234490, Biolegend, USA) and inflammatory factor expression of MHC-2, TLR4 were calculated as previously described.

For ELISA assay, RAW cells were cultured with 100 μg mL^-1^ NP, CMNP or EMNP under the condition of 100 ng mL^-1^ LPS for 6 h. Then collected the supernatants and centrifuged at 16000 g, 4 °C for 15 min for ELISA examination referring to the manual instructions of the kit (Bioswap, China).

### In vivo osteomyelitis model

The *in vivo* experiments were approved by the Ethics Committee of Animal Use of the Institute of Biomedical Sciences (No. 69/2017). The osteomyelitis model was conducted in Kunming mice (8 weeks old, female) according to previous reports with some modifications 46. After anesthetized, shaved and disinfected, a small incision along the tibia was made. After exposing the tibia through blunt dissection, a 1mm diameter bone defect was created by trephination. Then 1 × 10^7^ CFU of MRSA were delivered into the intramedullary canal through the bone defect. After that, injected 100 μg of NP, CMNP and EMNP into the defect. Carefully closed the muscle and skin. The tibia area of each mouse received ultraviolet irradiation (1 W cm^-2^, 90 s) treatment respectively. For positive control, vancomycin was injected intraperitoneally once (15 mg/kg).

For in vivo biocompatibility, the osteomyelitis mice with EMNP treatment after ultraviolet irradiation (1 W cm^-2^, 90 s) were sacrificed after 7 days. Hearts, lungs, spleens, lungs, kidneys were harvested for histological analysis.

For antibacterial analysis, the tibia was collected after euthanasia at day 5, the antibacterial effects were analyzed by extracted and homogenized the tibia in sterile PBS (1 mL) for standard plate counting.

For histological analysis, tibia was extracted for 5 days and 4weeks, and were fixed in 4% PFA. After decalcified for about 4 weeks with 10% EDTA, samples were embedded in paraffin wax and sectioned at 4 μm. Hematoxylin and eosin (H&E), Masson trichrome stains were performed according to manufacture protocols.

### Statistical analysis

All data were presented as means with standard deviations (SD). The significance analysis between two groups was performed through paired-t-test (*p < 0.05, **p < 0.01, ***p < 0.0001) with GraphPad Prism software (8.0).

## Supplementary Material

Supplementary figures and table.

## Abbreviations

MNPs: membrane-coated nanoparticles
NPs: nanoparticles
TCP: tricalcium phosphate
PAA-Ca: polyacrylic acid and Ca^2+^ ion complex
IPA: isopropyl alcohol
FTT: Ca_3_(PO4)_2_-Fe_3_O_4_@TiO_2_
FTIR: Fourier-transform infrared spectroscopy
XRD: X-ray diffraction
HA: hydroxyapatite
TEM: transmission electron microscope
DOPC: dioleoylphosphocholine
DPPC: dipalmitoyl-phosphatidylcholine
PRPs: pattern recognition receptors
PAMPs: pathogen-associated molecular patterns
RANKL: receptor activator of nuclear factor kappa-Β ligand
OPG: osteoprotegerin
TLR4: toll-like receptor 4
FITC: fluorescein isothiocyanate
LTA: lipoteichoic acid
LPS: lipopolysaccharides
S. aureus: staphylococcus aureus
E. coli: Escherichia coli
MRSA: methicillin-resistant staphylococcus aureus
PI: propidium iodide
ROS: reactive oxygen species
MHC-2: histocompatibility complex-2
IL-1: interleukin-1
IL-6: interleukin-6
H&E: hematoxylin and eosin
ICP-AES: inductively coupled plasma-atomic emission spectrometry
