# An electroporation strategy to synthesize the membrane-coated nanoparticles for enhanced anti-inflammation therapy in bone infection: Erratum

**DOI:** 10.7150/thno.121003

**Published:** 2025-09-15

**Authors:** Miusi Shi, Kailun Shen, Bin Yang, Peng Zhang, Kangle Lv, Haoning Qi, Yunxiao Wang, Mei Li, Quan Yuan, Yufeng Zhang

**Affiliations:** 1 State Key Laboratory Breeding Base of Basic Science of Stomatology (Hubei-MOST) and Key Laboratory of Oral Biomedicine, Ministry of Education, School and Hospital of Stomatology, Wuhan University, Wuhan 430079, China; 2 Key Laboratory of Catalysis and Energy Materials Chemistry of Ministry of Education & Hubei Key Laboratory of Catalysis and Materials Science, College of Resources and Environmental Science, South-Central University for Nationalities, Wuhan 430074, China; 3 Key Laboratory of Biomedical Polymers of Ministry of Education, College of Chemistry and Molecular Sciences, Wuhan University, Wuhan 430072, China; 4 Medical Research Institute, School of Medicine, Wuhan University, Wuhan, 430071, China

In the original version of the Supplementary Material, the graph of bacterial colony assay in Figure S16A (10 μg/mL group) was misplaced. The correct Figure S16 was attached below. The related quantification result was not affected since the data were collected directly from original file. Therefore, the correction does not alter any findings and conclusions of this work. The authors apologize for any inconvenience caused. The original experiment data with labels on the plate could be provided if required.

## Figures and Tables

**Figure A:**
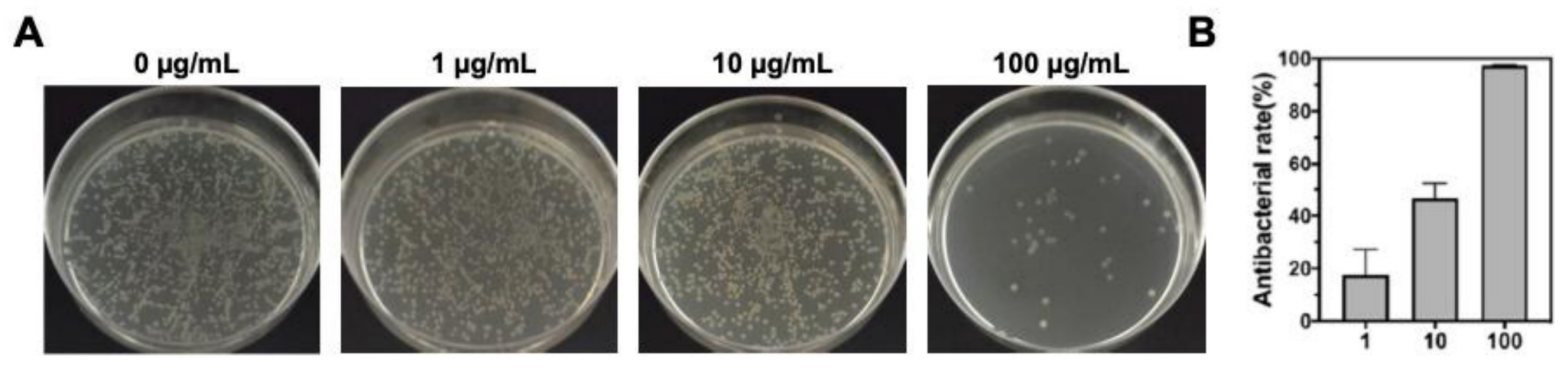
Originally Figure S16. (A) Bacteria colonies formation and (B) relative antibacterial rate of MRSA incubated with different dose of ampicillin.

