# Supplementary material for: An electroporation strategy to synthesize the membrane-coated nanoparticles for enhanced anti-inflammation therapy in bone infection
Source: Theranostics. 2021 Jan 1;11(5):2349–63. doi: 10.7150/thno.48407 (PMC7797679; doi:10.7150/thno.48407)
Supplement: Supplementary file 1 — Supplementary figures and table. [file thnov11p2349s1.pdf]

Supplementary Materials

An electroporation strategy to synthesize the membrane-coated nanoparticles for enhanced anti-inflammation therapy in bone infection

Miusi Shi[1,#], Kailun Shen[1,#], Bin Yang[2], Peng Zhang[1], Kangle Lv[2], Haoning Qi[1], Yunxiao Wang[1], Mei Li[2], Quan Yuan[3], Yufeng Zhang[1,4,*]

1. State Key Laboratory Breeding Base of Basic Science of Stomatology (Hubei-MOST) and Key Laboratory of Oral Biomedicine, Ministry of Education, School and Hospital of Stomatology, Wuhan University, Wuhan 430079, China
2. Key Laboratory of Catalysis and Energy Materials Chemistry of Ministry of Education & Hubei Key Laboratory of Catalysis and Materials Science, College of Resources and Environmental Science, South-Central University for Nationalities, Wuhan 430074, China
3. Key Laboratory of Biomedical Polymers of Ministry of Education, College of Chemistry and Molecular Sciences, Wuhan University, Wuhan 430072, China
4. Medical Research Institute, School of Medicine, Wuhan University, Wuhan, 430071, China

[#]These authors contributed equally.

*__Corresponding author:__

E-mail: zyf@whu.edu.cn, Tel/Fax: 86-027-87686267; (Yufeng Zhang)

Supplementary Figures

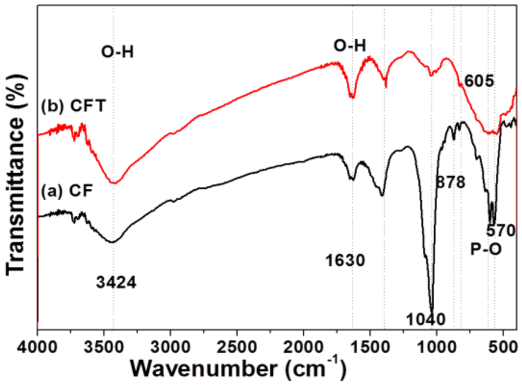

Figure S1. FTIR spectra of Fe_3O_4@TCP and TiO_2@Fe_3O_4@TCP.

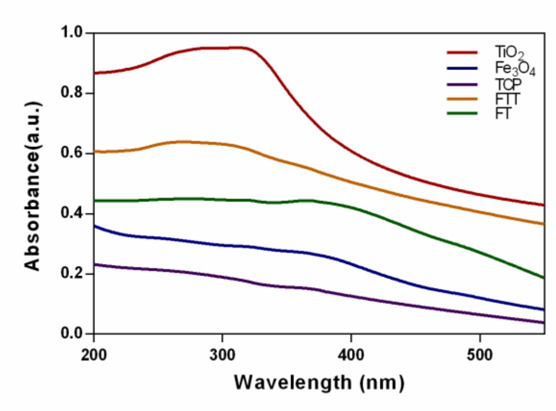

Figure S2. UV-vis absorption spectroscopy of TiO_2, Fe_3O_4, TCP, Fe_3O_4@TCP and TiO_2@Fe_3O_4@TCP.

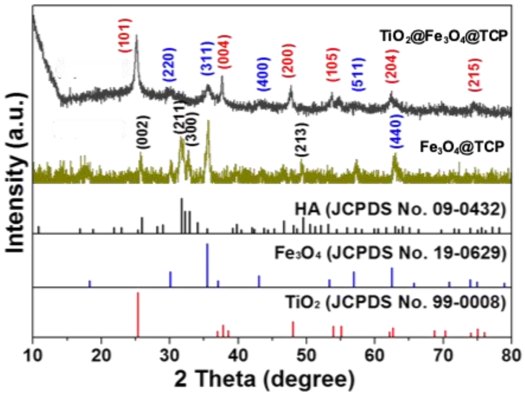

Figure S3. XRD patterns of TiO_2, Fe_3O_4, TCP, Fe_3O_4@TCP and TiO_2@Fe_3O_4@TCP.

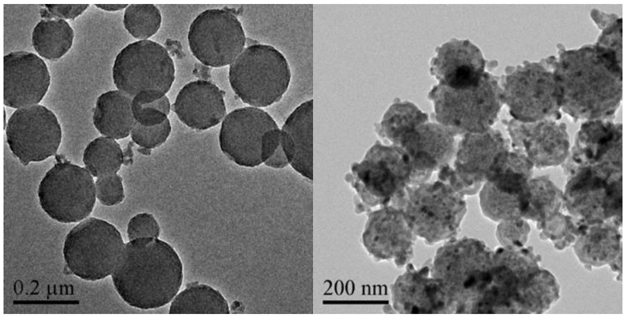

Figure S4. TEM images of Fe_3O_4@TCP (left) and TiO_2@Fe_3O_4@TCP (right).

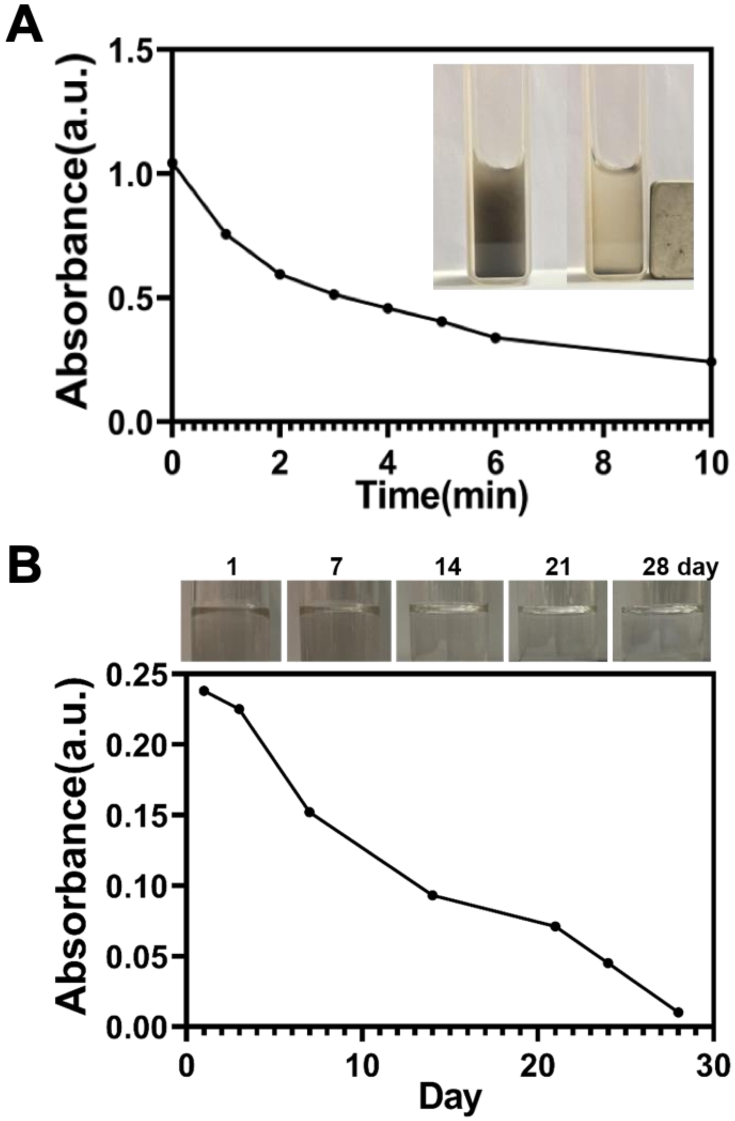

Figure S5. Magnetic test of $TiO_2@Fe_3O_4@TCP$(A) and longtime degradation (B).

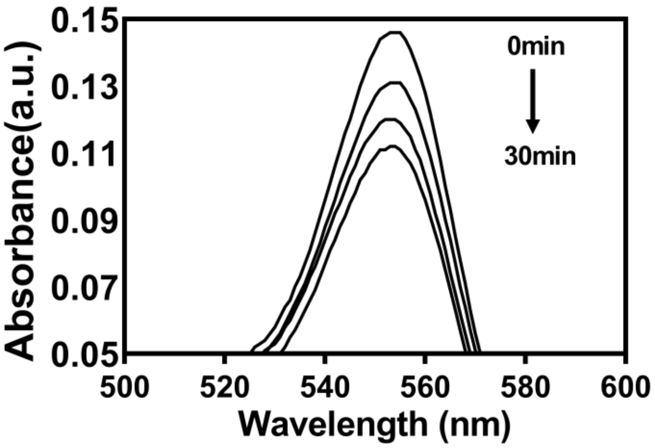

Figure S6. ROS releasing test of $TiO_2@Fe_3O_4@TCP$ by Rhodamine B degradation.

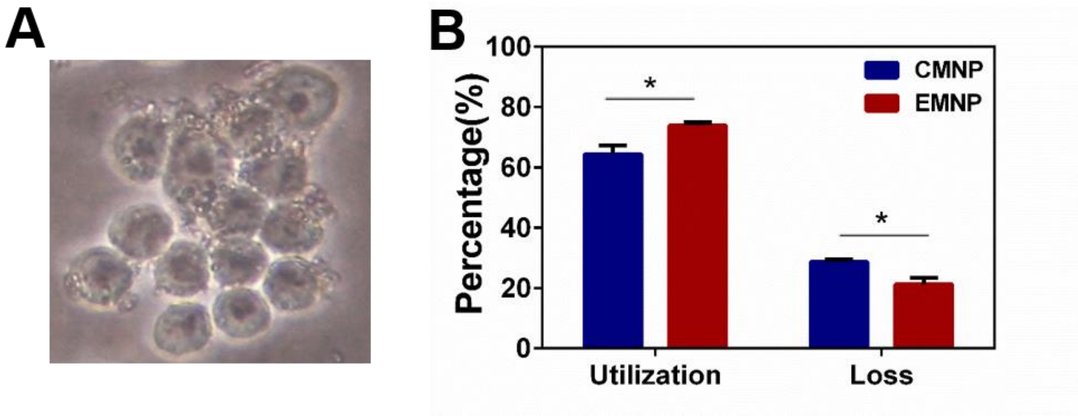

Figure S7. (A) Optical microscopy image of RAW 264.7 cells internalized the NP. (B) Ratio of membrane protein utilization and loss for the CMNP and EMNP (*$p < 0.05$).

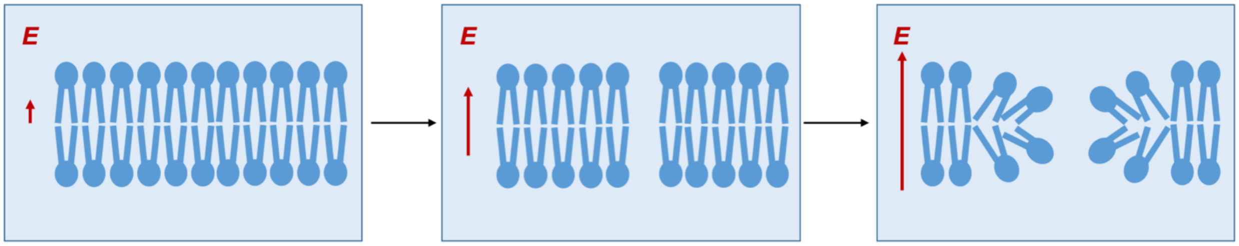

Figure S8. Conceptual scheme of molecular-level of mechanisms of hydrophilic pores formation under electrical field. Firstly, water molecules penetrate the bilayer and form an unstable hydrophobic pore (middle). Then adjacent lipid reorient their polar head groups toward the molecules to form a metastable hydrophilic pore (right).

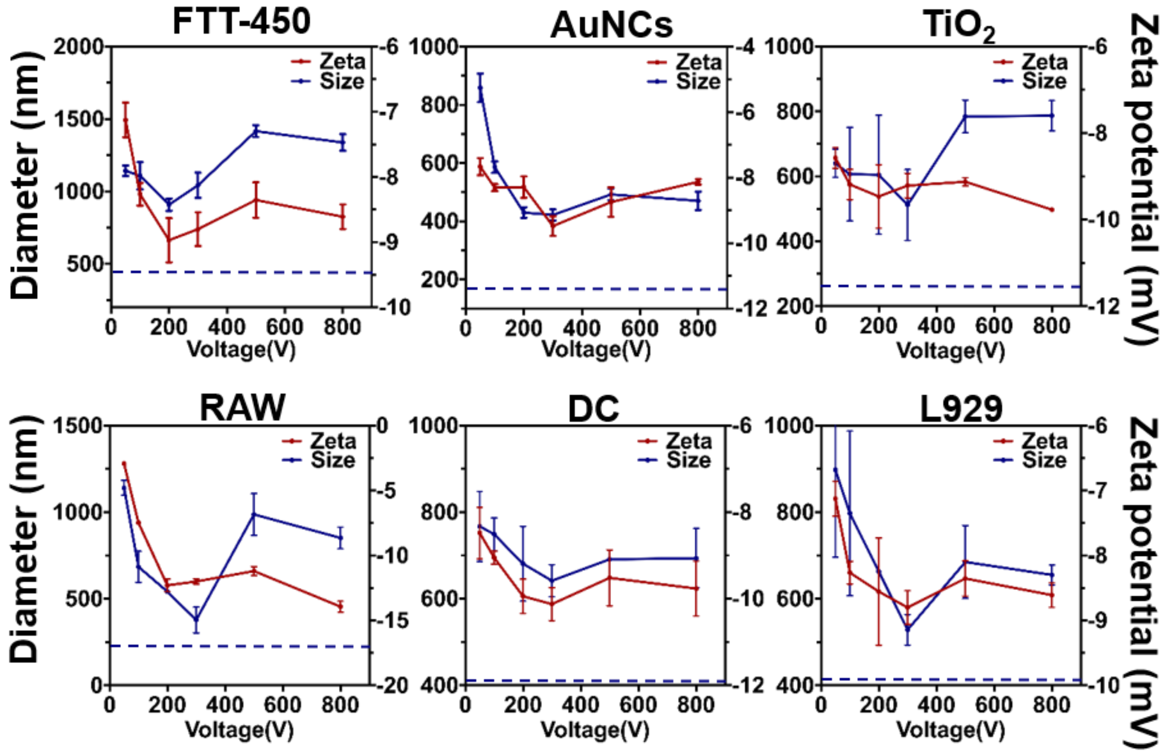

Figure S9. Diameters and zeta potentials of obtained EMNPs from varies nanoparticles (FTT-450, AgNCs, TiO_2) or cells (RAW, DC, L929) under different voltages. Blue dot lines represented the origin zeta potentials of nanoparticles.

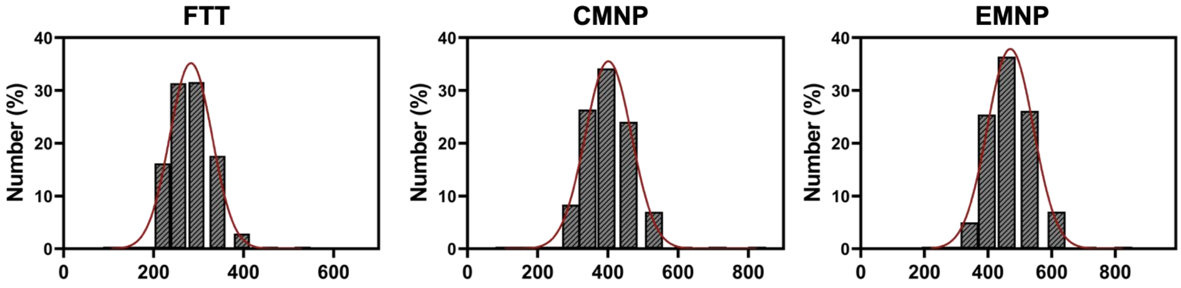

Figure S10. Size distribution of FTT, CMNP and EMNP using dynamic light scattering. PDI of FTT= 0.069, PDI of CMNP= 0.04, PDI of EMNP= 0.088.

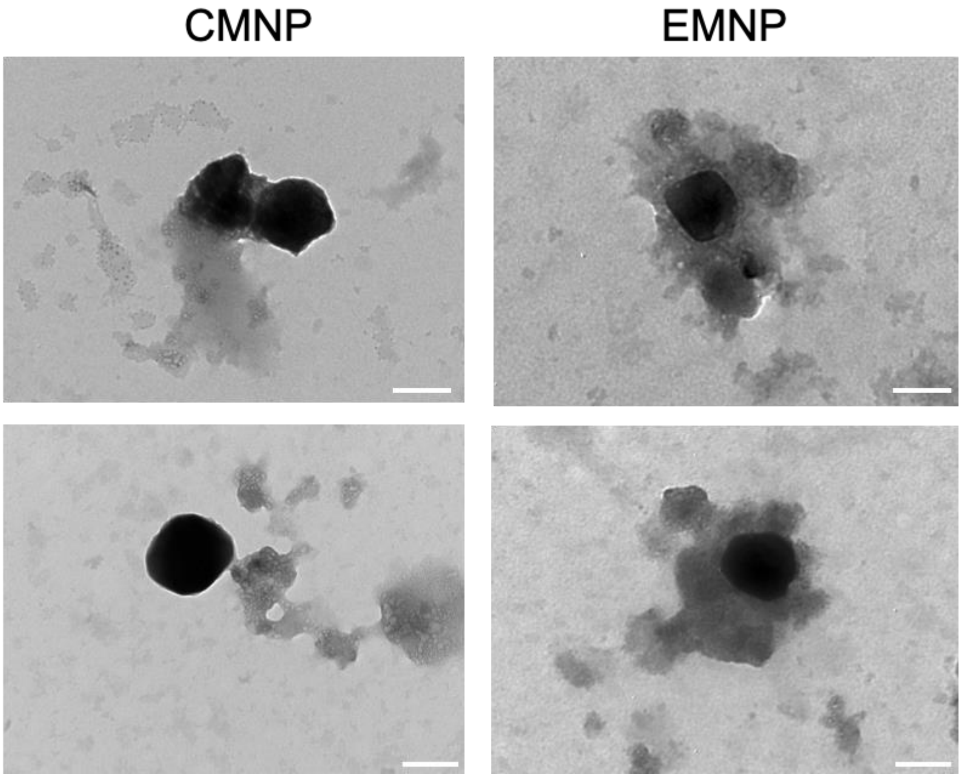

Figure S11. TEM images of membrane coating nanoparticles. Scale bar= 200 nm.

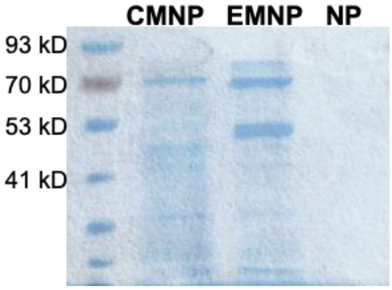

Figure S12. SDS-PAGE image of NP, CMNP and EMNP under same mass loading.

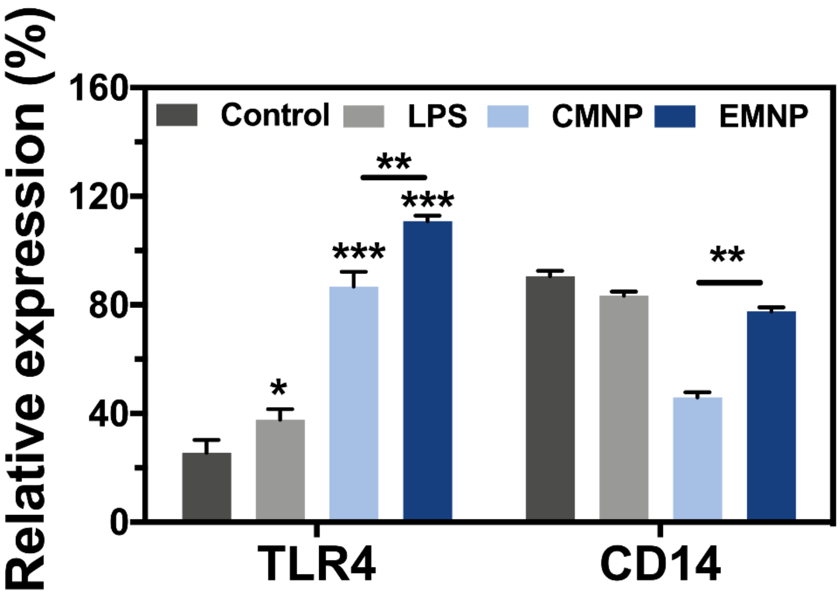

Figure S13. Quantification of TLR4, CD14 expression on RAW 264.7 cells, CMNP and EMNP with the stimulation of LPS. *p < 0.05, **p<0.01, ***p<0.0001.

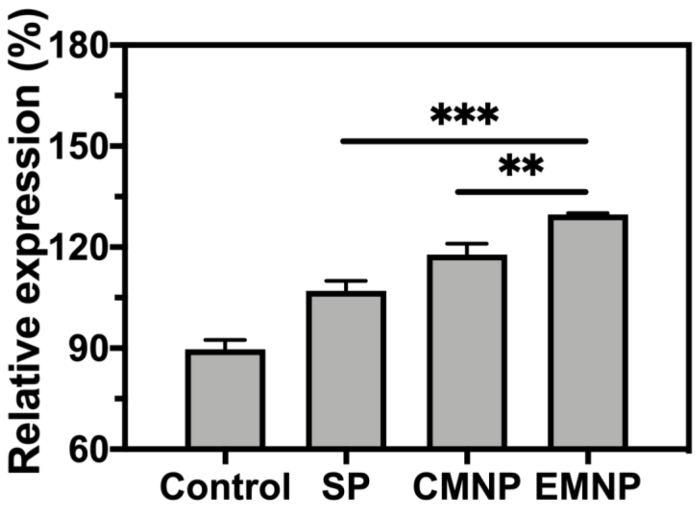

Figure S14. Quantification of TLR2 expression on RAW 264.7 cells, CMNP and EMNP with or MRSA supernatant. **p<0.01, ***p<0.0001.

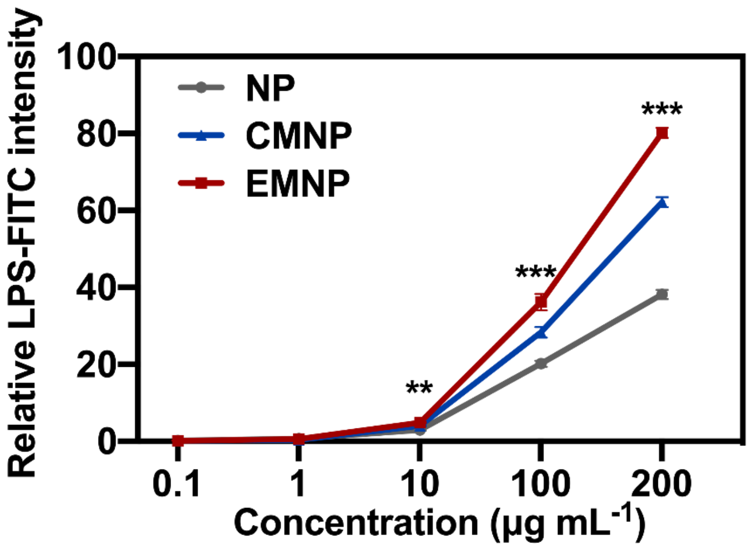

Figure S15. LPS-FITC intensity detection of LPS adsorption by NP, CMNP and EMNP at different initial concentrations of LPS (**p<0.01, ***p<0.0001).

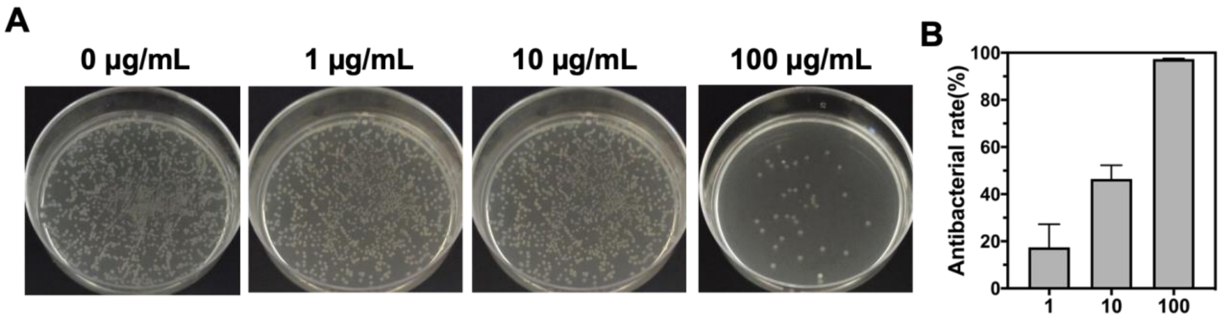

Figure S16. (A) Bacteria colonies formation and (B) relative antibacterial rate of MRSA incubated with different dose of ampicillin.

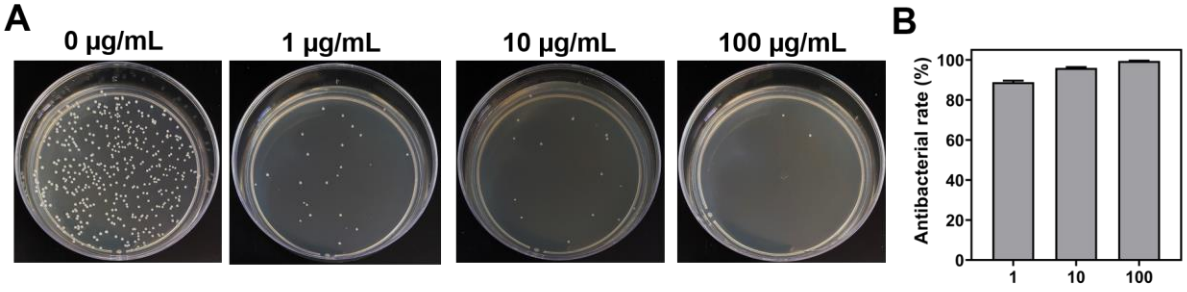

Figure S17. (A) Bacteria colonies formation and (B) relative antibacterial rate of MRSA incubated with different dose of vancomycin.

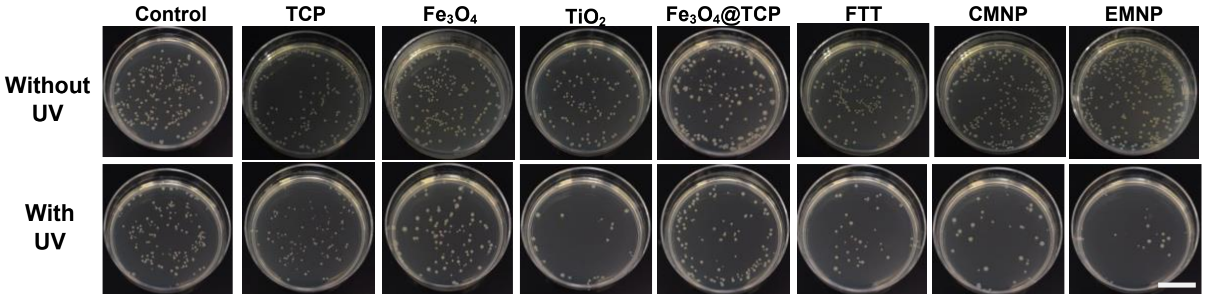

Figure S18. Bacteria colonies formation photos of MRSA incubated with TiO_2, Fe_3O_4, TCP, Fe_3O_4@TCP, TiO_2@Fe_3O_4@TCP, CMNP and EMNP with or without ultraviolet irradiation (1 W/ cm^2, 5 min). Scale bar = 2 cm. Relative quantification was shown in Figure 3C.

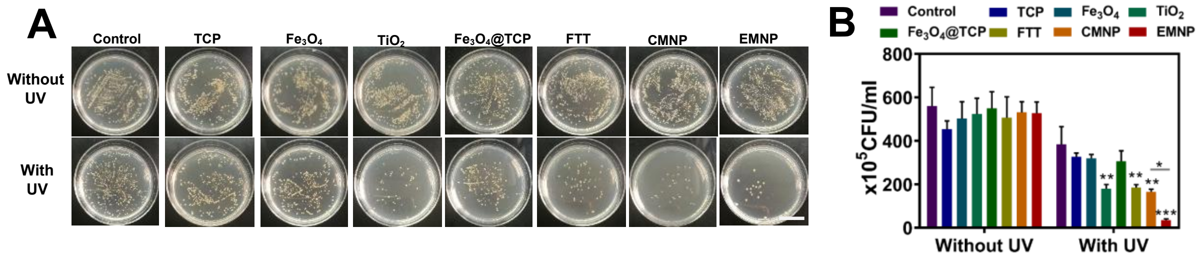

Figure S19. (A) Bacteria colonies formation and (B) quantitative analysis of *S.aureus* incubated with TiO_2, Fe_3O_4, TCP, Fe_3O_4@TCP, TiO_2@Fe_3O_4@TCP, CMNP and EMNP with or without ultraviolet irradiation (1 W/cm^2, 5 min) (scale bar = 2 cm, $*p < 0.05$, $**p < 0.01$, $***p < 0.0001$).

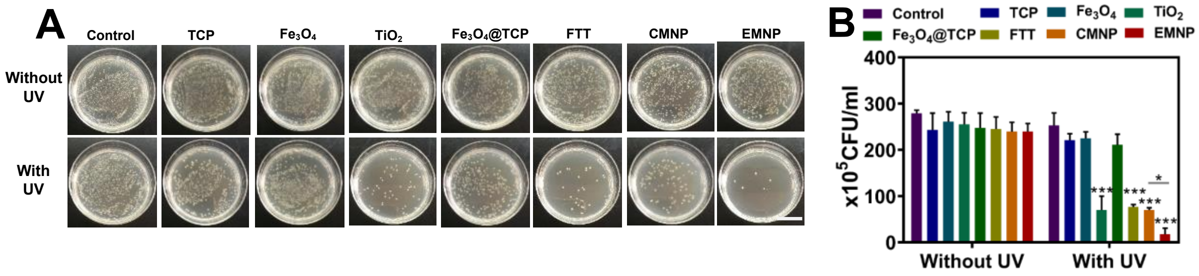

Figure S20. (A) Bacteria colonies formation and (B) quantitative analysis of *E.coli* incubated with TiO_2, Fe_3O_4, TCP, Fe_3O_4@TCP, TiO_2@Fe_3O_4@TCP, CMNP and EMNP with or without ultraviolet irradiation (1 W/cm^2, 5 min) (scale bar = 2 cm, *$p < 0.05$, ***$p < 0.0001$).

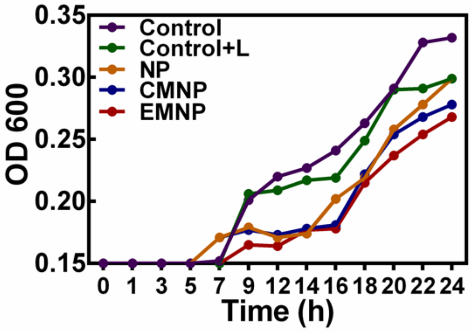

Figure S21. Growth curves of MRSA incubated with NP, CMNP and EMNP with or without ultraviolet irradiation (1 W/cm^2, 5 min)

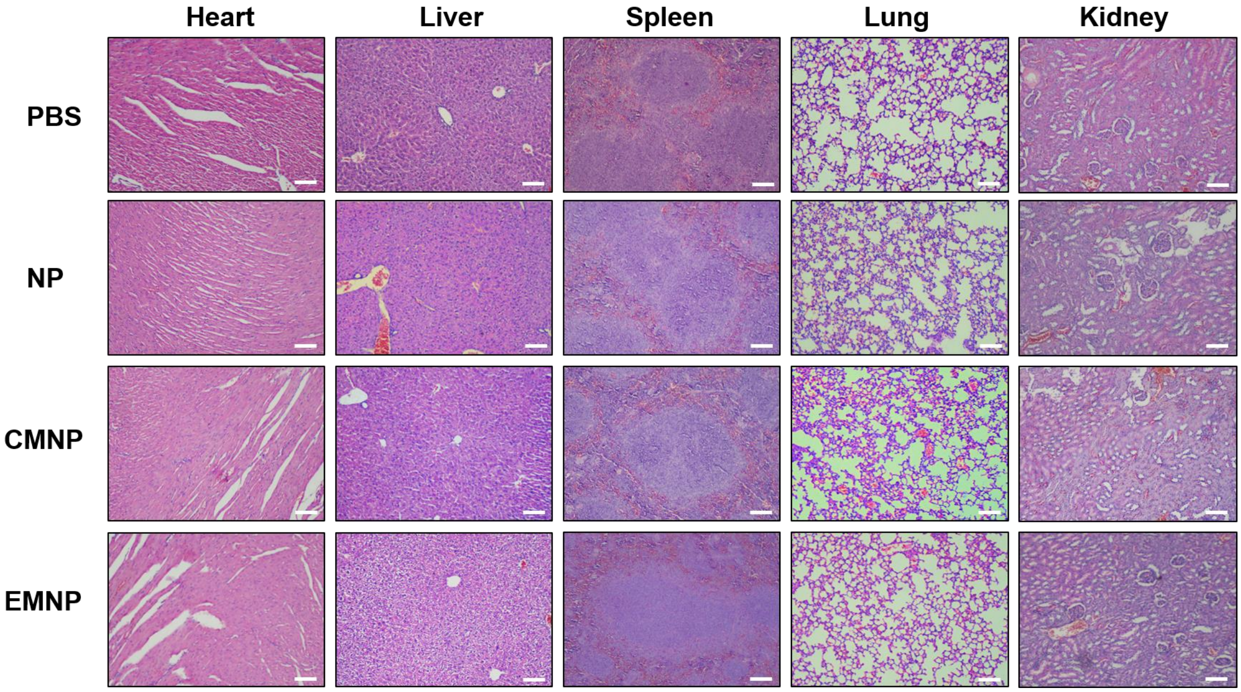

Figure S22. HE staining of heart, liver, spleen, lung and kidney stained after 7 days of treatment with PBS, NP, CMNP, EMNP. Scale bar = 200 μm.

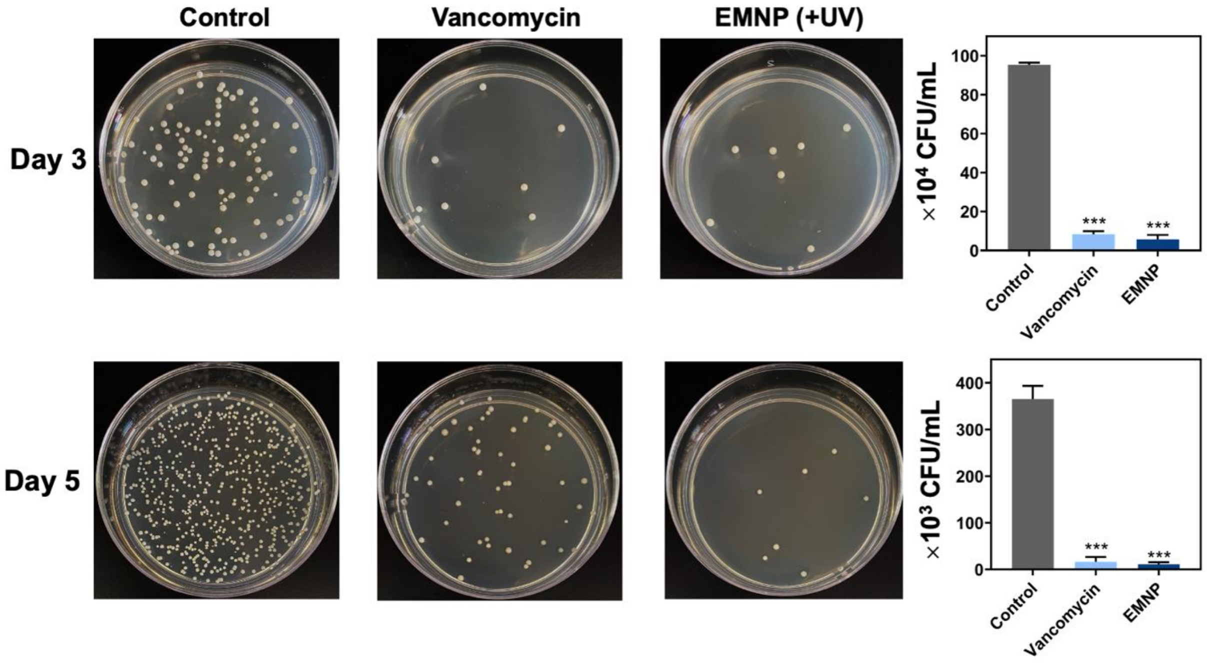

Figure S23. Photos and quantification of the bacterial colony of the femur bone tissues treated with vancomycin and EMNP with ultraviolet irradiation after 3 or 5 days (***$p < 0.0001$).

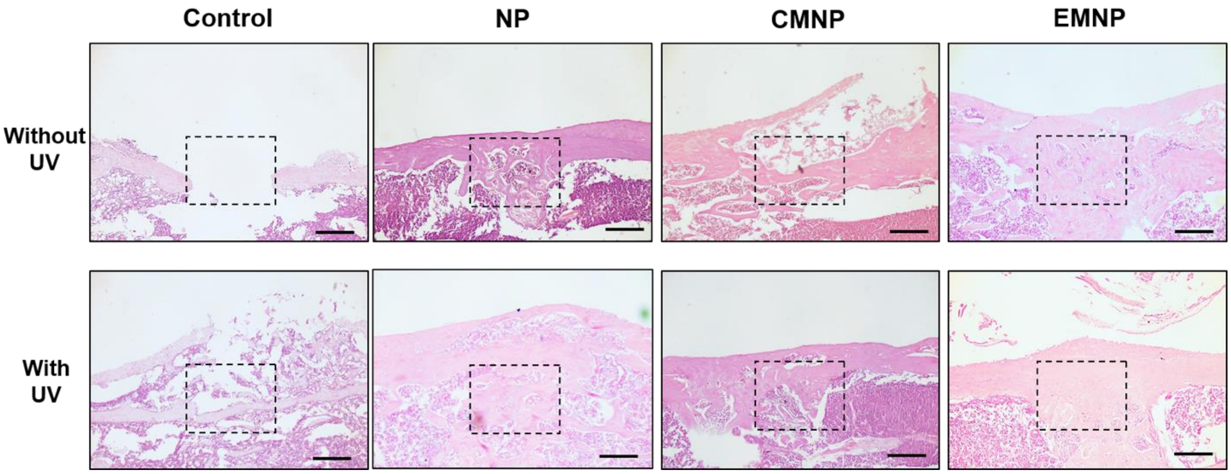

Figure S24. H&E staining of bone repair area 4 weeks after treatment (black dot line showed the bone defect area after operation, scale bar = 100 μm).

Supplementary Table

Table 1 Primer and probe sequences for PCR genes.

Names	Forward	Reverse
IL-1β	TGGAGAGTGTGGATCCCAAG	GGTGCTGATGTACCAGTTGG
IL-6	ATAGTCCTTCCTACCCCAATTTCC	GATGAATTGGATGGTCTTGGTCC
GAPDH	GCACCGTCAAGGCTGAGAAC	TGGTGAAGACGCCAGTGGA
